# Lifestyle Modifiers of Non-Infectious Uveitis: A Narrative Review of Current Evidence and Future Directions

**DOI:** 10.3390/jpm16100511

**Published:** 2026-09-30

**Authors:** Priya D. Samalia, Serena S. Chen, Lucy M. Lu, Joanne L. Sims, Karen Lindsay, Rachael L. Niederer

**Affiliations:** 1 Monash Health, Melbourne, VIC 3168, Australia; 2 Department of Ophthalmology, Grafton Campus, University of Auckland, Auckland 1023, New Zealand; 3 Health New Zealand Auckland, Auckland 1051, New Zealand

**Keywords:** gut-eye axis, immune modulation, inflammation, lifestyle medicine, non-infectious uveitis

## Abstract

**Background:** Non-infectious uveitis is a leading cause of preventable visual impairment and arises from complex interactions between genetic susceptibility, environmental exposures, and immune dysregulation. **Purpose:** To review current evidence regarding modifiable lifestyle factors that may influence the development, severity, recurrence, and outcomes of uveitis. This narrative review used structured searches of MEDLINE, PubMed, Embase and the Cochrane Database of Systematic Reviews from database inception, last updated on 30 June 2026, supplemented by reference-list screening. **Results:** Diet, smoking, obesity, physical activity, sleep, psychological stress, alcohol consumption, and micronutrient status may affect uveitis through effects on systemic inflammation, Th1/Th17 immunity, regulatory T-cell function, oxidative stress, and the gut microbiome. **Conclusions:** Although the evidence remains limited and largely observational, lifestyle assessment may support personalized adjunctive care by considering the patient’s uveitis phenotype, systemic comorbidities, treatment exposures and individual priorities. Lifestyle measures should support systemic health and shared decision-making without being presented as substitutes for established non-infectious uveitis treatment or as proven ocular disease-modifying interventions.

## 1. Introduction

**Figure 1:**
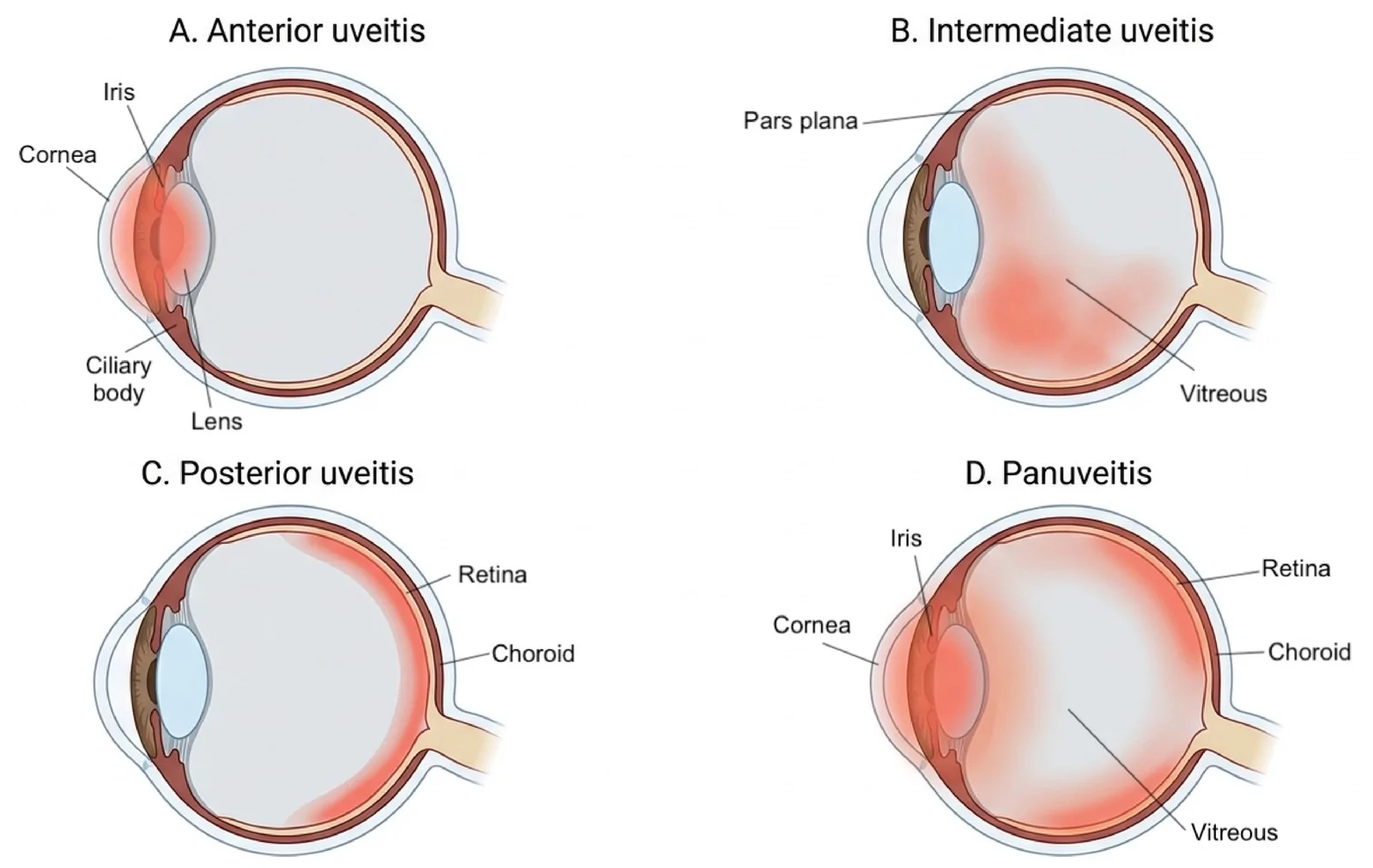
Anatomical classifications of uveitis according to the predominant site of inflammation. Uveitis is classified anatomically as anterior uveitis, intermediate uveitis, posterior uveitis or panuveitis. This anatomical classification describes the principal site of inflammation and does not determine whether the underlying etiology is infectious or non-infectious.

Uveitis encompasses a heterogeneous group of intraocular inflammatory disorders affecting the uveal tract (iris, ciliary body and choroid) and adjacent intraocular structures. It remains a major cause of visual impairment and preventable blindness worldwide, particularly among working-age adults [1,2]. It is classified anatomically as anterior, intermediate, posterior, or panuveitis according to the predominant site of inflammation (Figure 1) and may be infectious or non-infectious in etiology. Non-infectious uveitis (NIU) is primarily immune-mediated and may occur in isolation or in association with systemic inflammatory diseases, including the spondyloarthropathies, sarcoidosis, Behçet disease, inflammatory bowel disease (IBD), and juvenile idiopathic arthritis (JIA) [3,4].

Despite being highly responsive to steroid treatment, and major advances in multimodal imaging and corticosteroid-sparing immunomodulatory therapy, many patients experience chronic or relapsing disease requiring prolonged treatment and monitoring. Visual impairment may result from persistent inflammation or its complications such as cystoid macular edema (CME), cataract, glaucoma, retinal vasculitis, epiretinal membrane (ERM), and choroidal neovascularization. Beyond visual morbidity, NIU is associated with impaired quality of life, psychological distress, work disability, and substantial healthcare utilization [4,5,6,7].

Growing evidence suggests that interactions between genetic predisposition and environmental exposures shape disease susceptibility, inflammatory activity, and treatment response across immune-mediated diseases. Lifestyle factors including smoking, diet, obesity, physical activity, sleep, psychological stress, alcohol consumption, and metabolic health influence shared biological pathways such as the gut microbiome, immunometabolism, oxidative stress, neuroendocrine signaling, and adaptive immune responses [4,8,9,10]. As many of these pathways are also implicated in the immunopathogenesis of NIU, lifestyle factors may contribute to inter-individual differences in disease expression and clinical outcomes, with their effects potentially varying according to the underlying uveitis subtype and disease-specific immune pathways [4,11]. Patients often enquire about lifestyle changes that may optimize their response to treatment [12,13].

This review synthesizes current evidence regarding modifiable lifestyle factors in NIU, focusing on their biological mechanisms, clinical evidence, and implications for patient management. We also highlight current knowledge gaps and examine how lifestyle evidence could inform personalized adjunctive care according to uveitis phenotype, systemic comorbidities, treatment regimen and patient priorities. In this context, personalized medicine involves identifying which recommendations are supported for a particular patient, which represent general health or treatment-safety advice, and which remain research hypotheses.

This narrative review updates and extends on an earlier review [14] by incorporating a broader range of lifestyle factors and recent human evidence concerning metabolic therapies, biologic drug exposure and psychological interventions, while distinguishing clinical associations from mechanistic evidence and general health advice.

## 2. Methods

This narrative review was developed collaboratively to examine potentially modifiable lifestyle factors in NIU. Each contributing author was responsible for identifying, reviewing and selecting literature for an assigned lifestyle domain and drafting the corresponding section. A common prospective search protocol was not specified at the outset, and the original domain-specific search strings were not retained.

The draft sections and their references were consolidated and synthesised by P.S. Duplicate citations in the combined reference lists were identified and removed manually. Study selection was not independently duplicated. All authors reviewed the complete manuscript and had the opportunity to comment on the selection and interpretation of evidence. No formal process for adjudicating eligibility decisions was specified; no disagreements about inclusion arose. Reference lists of included articles and relevant systematic reviews were also examined for additional publications.

To document the search approach during revision, searches of MEDLINE via Ovid, PubMed, Embase via Ovid, and the Cochrane Database of Systematic Reviews were run on 27 September 2026 without a publication-year restriction. The searches used controlled vocabulary, where available, and free-text terms covering uveitis and the lifestyle domains addressed in the review. Supplementary Table S1 reports these documented search strategies, dates and results. Publications dated after 30 June 2026 were outside the review’s inclusion period, although they may appear in the search-result counts in Table S1.

Eligible publications included peer-reviewed English-language human observational and interventional studies, experimental animal and in vitro studies, and systematic reviews relevant to the lifestyle domains. Single case reports, conference abstracts without an available full-text publication, and non-English-language publications were excluded. Direct human evidence in NIU was prioritised. Experimental uveitis models and mechanistic studies were included to examine biological plausibility. Evidence from other immune-mediated diseases was considered when direct NIU evidence was absent or limited and the condition offered relevant information about shared inflammatory pathways, systemic manifestations, treatment exposure, or broader health effects. Such conditions were selected for their immunological or clinical relevance to NIU, rather than through a separate systematic search of every immune-mediated disease. Extrapolated evidence was identified as indirect and was not treated as evidence of an ocular treatment effect.

Findings were synthesised narratively by lifestyle domain because populations, uveitis phenotypes, exposures, comparators and outcomes varied substantially. No meta-analysis was undertaken. The principal human uveitis studies were considered in relation to study design, sample size, exposure and outcome ascertainment, adjustment for confounders, precision, temporality and applicability to NIU. Supplementary Table S2 summarises their populations and phenotypes, sample sizes, exposures, comparators, outcomes, adjusted estimates and confidence intervals where reported, and main limitations. The synthesis distinguishes human NIU associations from experimental findings, evidence extrapolated from other diseases, and evidence that modifying an exposure improves ocular outcomes.

## 3. Shared Biological Pathways Linking Lifestyle Factors and Non-Infectious Uveitis

**Figure 2:**
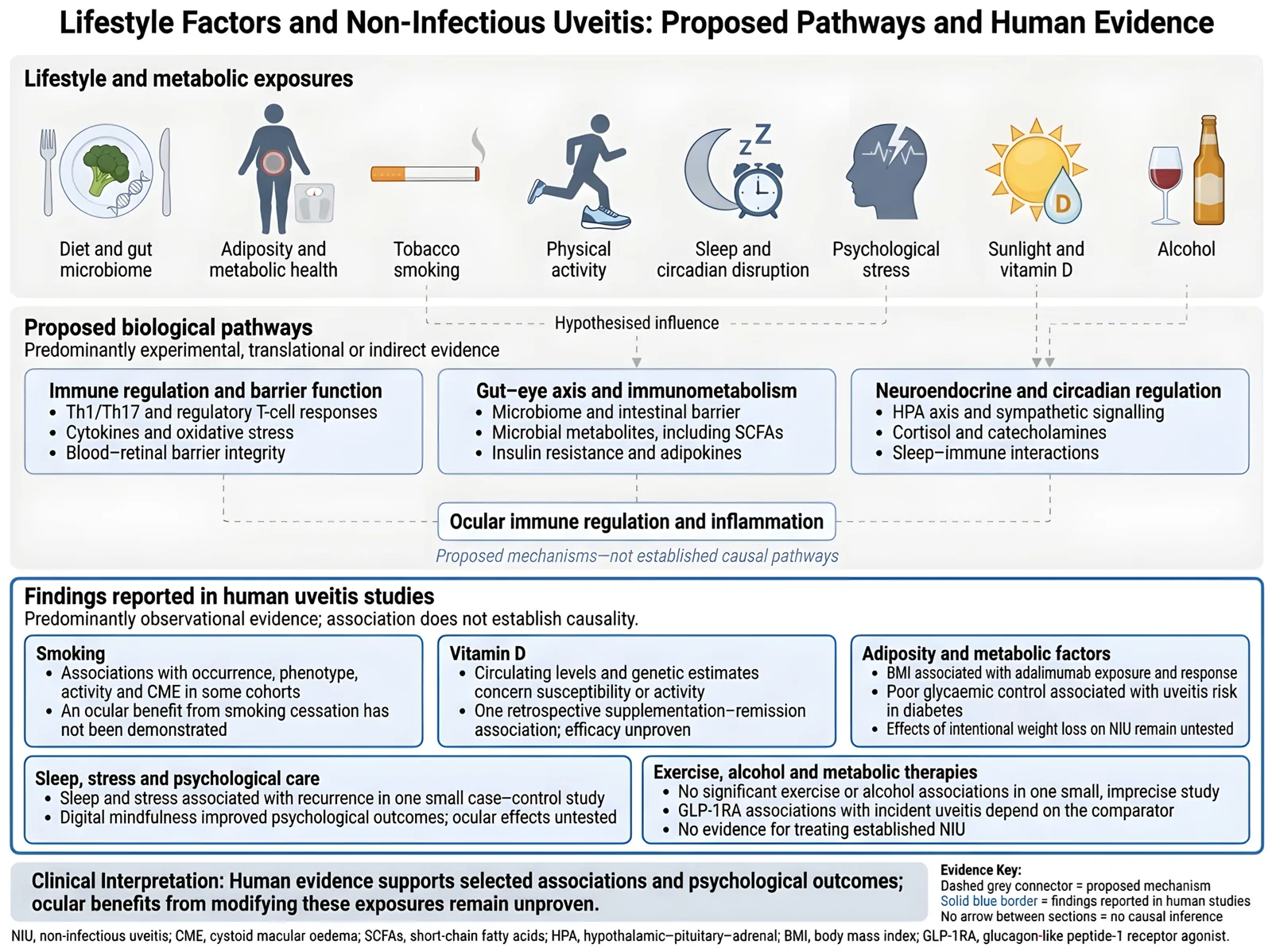
Proposed biological pathways and human evidence relevant to non-infectious uveitis (NIU). The upper panels show hypothesised pathways supported predominantly by experimental, translational or indirect evidence. Dashed grey connectors indicate proposed influences, not established causal effects. The blue-outlined lower panel summarises findings from human uveitis studies, including studies relevant to NIU. These associations and psychological outcomes do not demonstrate that modifying lifestyle exposures prevents or treats NIU.

Direct evidence linking lifestyle factors to NIU remains limited. However, experimental autoimmune uveitis (EAU), translational studies and evidence from related immune-mediated diseases identify several interconnected pathways through which behavioral and environmental exposures could influence ocular inflammation (Figure 2). These include immune dysregulation, the gut-eye axis, immunometabolic and oxidative processes, and neuroimmune signaling [4,8,9,15]. Together, they provide biological plausibility but do not establish that modifying these exposures alters NIU susceptibility, activity, treatment response or long-term outcomes.

### 3.1. Immune Dysregulation

NIU involves disruption of ocular immune homeostasis and immune privilege. Under physiological conditions, the blood-retina and blood-aqueous barriers, regulatory T cells (Treg), anti-inflammatory cytokine networks and local immunosuppressive mechanisms maintain ocular immune tolerance. Their disruption permits inflammatory-cell recruitment and persistent intraocular inflammation. Several NIU phenotypes are characterized by an imbalance between pathogenic T helper 1 (Th1) and T helper 17 (Th17) responses and Treg-mediated regulation, accompanied by pro-inflammatory cytokine production and barrier dysfunction [4,11]. Lifestyle-related exposure may influence these pathways, although their clinical effects in NIU remain uncertain.

### 3.2. Gut–Eye Axis and Immunometabolism

The gut microbiome regulates systemic immune homeostasis and has been implicated in experimental and human uveitis. Changes in microbial composition, intestinal barrier integrity and microbial metabolites, including short-chain fatty acids (SCFAs), may alter immune regulation and systemic inflammatory activity [15,16,17,18]. These processes intersect with immunometabolic health: obesity and insulin resistance are associated with adipokine dysregulation, oxidative stress, endothelial dysfunction and chronic low-grade inflammation [19,20]. Diet, physical activity and body composition may therefore influence overlapping microbial, metabolic and inflammatory pathways. However, whether alterations in the gut-eye axis contribute causally to NIU or modify its clinical course remains uncertain.

### 3.3. Neuroimmune Regulation

Sleep, psychological stress and circadian disruption influence immune homeostasis through interactions among the hypothalamic–pituitary–adrenal axis (HPA), sympathetic nervous system and immune system [21,22,23,24,25,26,27]. Dysregulation of these pathways may alter glucocorticoid signaling, immune-cell trafficking, cytokine production and gastrointestinal microbial and barrier function [28,29,30]. These relationships are bidirectional: neuroendocrine disturbance can influence inflammation, while inflammatory signaling can affect sleep, mood and stress responses. Sleep and psychological factors could therefore interact with systemic inflammatory activity in NIU, although direct clinical evidence remains limited.

## 4. Smoking and Tobacco Exposure

Smoking is among the most consistently studied modifiable exposures in uveitis. Observational associations have been reported with disease occurrence, inflammatory activity, treatment response and visual outcomes, although findings vary across uveitis phenotypes and do not establish that cessation alters ocular outcomes.

### 4.1. Biological Mechanisms

Beyond the shared pathways outlines in Section 3, smoking exposes tissue to reactive oxygen species and other toxic compounds that can overwhelm antioxidant defenses, damage DNA and mitochondria, and activate redox-sensitive inflammatory signaling [31,32,33,34,35,36,37,38]. Tobacco exposure may also reprogram innate and adaptive immune cells, alter cytokine production and regulatory function, and activate the NLRP3 inflammasome [31,33,34,35,37,39,40,41].

Smoking-related endothelial dysfunction and loss of vascular-barrier integrity may facilitate inflammatory-cell trafficking into immune-privileged tissue [37,40,42]. Persistent epigenetic changes, including altered DNA methylation and histone modification, may further modify subsequent immune responses through trained innate immunity [37,38]. Second-hand smoking has also been associated with dose-dependent choroidal thinning, suggesting a possible additional contribution from ocular vascular and structural injury [43]. However, these mechanisms do not establish that smoking directly causes or worsens a particular NIU phenotype.

### 4.2. Clinical Evidence

Observational studies consistently report an association between smoking and uveitis, although the populations, phenotypes, smoking definitions, and outcomes vary. In a recent systematic review and meta-analysis, Kuč et al. included seven observational studies: four retrospective case–control studies, one retrospective cohort study and two cross-sectional studies [44]. Four retrospective studies contributed to the primary meta-analysis, comprising 1557 individuals with greater odds of uveitis (pooled OR 1.97; 95%CI 1.71–2.27; *I*^2^ = 46%). Former smoking was also associated with uveitis (pooled OR 1.23; 95% CI 1.05–1.44), although heterogeneity was high (*I*^2^ = 79%). These findings support an association between smoking exposure and uveitis but do not establish causality or demonstrate that smoking cessation changes subsequent ocular outcomes. The pooled studies were observational and varied in uveitis definition and phenotype, with some including infectious uveitis or presumed ocular histoplasmosis syndrome.

Lin et al. conducted a retrospective case–control study comparing 564 patients with ocular inflammation with 564 comprehensive eye-clinic controls [33]. The case population included infectious, non-infectious, and idiopathic ocular inflammation. Compared with controls who had never smoked, a history of current or previous smoking was associated with ocular inflammation overall (adjusted OR 2.2; 95% CI 1.7–3.0), including both infectious uveitis (OR 4.5; 95% CI 2.3–9.0) and non-infectious uveitis (OR 2.1; 95% CI 1.6–2.8). Associations were also reported for anterior uveitis (OR 1.7; 95% CI 1.2–2.4), intermediate uveitis (OR 2.7; 95% CI 1.4–5.6), posterior uveitis (OR 3.2; 95% CI 1.3–7.9), and panuveitis (OR 3.9; 95% CI 2.4–6.1). These anatomical analyses were not restricted to NIU.

Lin et al. also found stronger associations between smoking and uveitis phenotypes complicated by CME. Relative to eye-clinic controls, smoking was associated with intermediate uveitis with CME (OR 8.4; 95% CI 2.5–28.8) and panuveitis with CME (OR 8.0; 95% CI 3.3–19.5), compared with weaker associations for the corresponding phenotypes without CME (OR 1.5; 95% CI 0.6–3.8 and OR 3.1; 95% CI 1.8–5.2, respectively) [33]. The wide confidence intervals reflect the small numbers of patients in the CME subgroups and indicate uncertainty regarding the magnitude of these associations. Nevertheless, the findings are consistent with other observational studies associating smoking with CME in intermediate uveitis and with a more complicated clinical course in NIU [34,45], but the available evidence remains vulnerable to confounding and does not establish that smoking cessation reverses these risks.

Other observational studies have associated smoking with younger presentation, greater inflammatory activity, more intensive corticosteroid treatment, slower achievement of quiescence, bilateral disease and poorer visual acuity [31,34,46]. In a retrospective series of 20 patients with intermediate uveitis treated with posterior sub-Tenon triamcinolone, Helm and Holland found a weak, non-statistically significant association between visual improvement and a history of not smoking after adjustment for age (*p* = 0.073) [47]. One cross-sectional study found numerically poorer vision-related quality of life among active smokers, but the association was not statistically significant and was not independently adjusted [48]. However, findings concerning recurrence, remission, ocular hypertension, posterior synechiae, and the requirement for second-line immunosuppression are inconsistent [34,49,50,51]. The evidence therefore supports smoking as a marker associated with NIU susceptibility and, in some populations, a more complicated disease course, but the magnitude and clinical relevance of these associations vary across uveitis phenotypes.

### 4.3. Special Considerations

Smoking cessation should be routinely recommended because of its established cardiovascular, respiratory, oncological, and general health benefits. However, no uveitis-specific cessation trial or longitudinal study has established that stopping smoking reduces ocular inflammatory activity, recurrence, CME, corticosteroid requirements, or treatment failure. The association between former smoking and uveitis reported by Kuč et al. should not be interpreted as evidence that cessation is ineffective, because the included studies did not evaluate cessation interventions or account consistently for cumulative exposure, time since cessation, or smoking intensity. Smoking cessation should therefore be presented as strongly justified general-health advice in the context of an adverse observational association with uveitis, rather than as a proven ocular disease-modifying intervention [44].

Evidence regarding passive smoking is limited, although second-hand smoke promotes systemic inflammation, endothelial dysfunction, oxidative stress, and retinal vascular dysfunction, providing possible mechanisms through which passive exposure may influence ocular inflammation [43,52].

Electronic cigarettes (e-cigarettes) and heat-not-burn tobacco products avoid many combustion-related toxins but generate aerosols containing nicotine, aldehydes, heavy metals, and ultrafine particulate matter capable of inducing oxidative stress, endothelial dysfunction, and immune activation [53]. Experimental studies further demonstrate epithelial barrier disruption, altered immune cell function, and increased pro-inflammatory cytokine signaling, indicating that vaping is not biologically inert [54,55].

Emerging observational evidence also suggests potential adverse effects in immune-mediated disease. E-cigarette use has been associated with an increased risk of incident uveitis [53,56], while studies in other immune-mediated diseases report increased odds of inflammatory arthritis and higher postoperative recurrence in Crohn’s disease among users of heat-not-burn products [57,58]. Although causality remains uncertain and not all studies have demonstrated adverse clinical outcomes [59], current evidence does not support vaping as a safe alternative for patients with NIU.

Smoking in Behçet disease warrants consideration. Although some studies suggest nicotine may improve mucocutaneous manifestations, observational studies have associated smoking with greater overall disease severity and ocular involvement, particularly in conjunction with HLA-B51 [60,61,62]. These findings are retrospective and do not establish that smoking causes ocular involvement. Given the conflicting disease-specific evidence and the established systemic harms of tobacco use, smoking cessation should continue to be recommended for patients with Behçet disease and other forms of NIU.

## 5. Diet and Nutrition

Growing experimental and clinical data support the potential role of diet and nutrition as an adjunct to conventional management. A summary of dietary interventions is shown in Table 1. 

### 5.1. Biological Mechanisms

Beyond the shared gut-eye pathways outlined in Section 3, diet may influence immune regulation by altering microbial composition, intestinal barrier function and microbiota-derived metabolites [18]. Fiber-rich diets promote production of SCFAs, particularly butyrate, which support barrier integrity, Treg-cell differentiation and immune tolerance [63,64,65,66,67]. Conversely, Western dietary patterns may promote dysbiosis, increased intestinal permeability and systemic inflammation [15,16,17,19,68,69,70,71]. Microbial alterations have been reported in HLA-B27-associated uveitis, Behçet disease, and Vogt-Koyanagi-Harada disease, while HLA-B27 and HLA-A29 may influence host-microbiome interactions [15,16,18,19,63,64]. These findings support a possible gene-microbiome-environment interaction but do not establish that dietary modification alters NIU.

### 5.2. Clinical and Experimental Evidence

Among dietary patterns, the Mediterranean diet has the strongest evidence for reducing systemic inflammation and the risk of immune-mediated disease, although it has not been directly evaluated in NIU [69]. Conversely, Western dietary patterns are associated with increased systemic inflammation, while high-fat diets exacerbate experimental autoimmune uveitis (EAU) through gut dysbiosis, impaired intestinal barrier function, and enhanced Th1/Th17 responses [14,68,69,71].

Several dietary interventions have demonstrated anti-inflammatory effects in EAU. Fermentable fiber attenuates disease by restoring intestinal barrier integrity, increasing microbiota-derived short-chain fatty acid production, and promoting Treg-cell responses [15,19,67]. Similarly, dietary omega-3 fatty acids reduce intraocular IFN-γ and IL-17 expression, suppress Th1/Th17 differentiation, and decrease disease severity [66,72,73].

Curcumin has also shown anti-inflammatory effects through modulation of NF-κB, Nrf2/HO-1, and IL-23/IL-17 signaling pathways [74,75,76]. Human evidence in uveitis is limited to heterogeneous, non-randomized studies using different formulations. An uncontrolled study of chronic anterior uveitis included 32 completers treated with curcumin 375 mg three times daily for 12 weeks; 18 received curcumin with topical mydriatic and 14 also received antitubercular therapy, with clinical improvement reported in both groups [77]. In recurrent anterior uveitis, an uncontrolled before-after study of 106 completers reported fewer relapses after 12 months of adjunctive phosphatidylcholine-complexed curcumin (Meriva 600 mg twice daily) alongside ongoing conventional therapy [78]. A retrospective study of 27 patients with JIA-associated uveitis reported reduced laser flare after one daily tablet of another phospholipid formulation added to systemic immunosuppression [79]. Finally, a prospective observational study of uveitic CME compared standard corticosteroid therapy alone in 11 patients with adjunctive CuruWIN 60 mg twice daily in 15 patients and reported greater improvements in visual acuity and central macular thickness [80]. Differences in formulation and dose, small samples, non-randomized allocation and predominantly uncontrolled designs preclude firm conclusions regarding efficacy.

Other interventions, including ketogenic diets, caloric restriction, intermittent fasting, green tea polyphenols, caffeine, and antioxidant or trace element supplementation, have shown anti-inflammatory or retinoprotective effects primarily in experimental models. However, evidence is insufficient to support their routine use in patients with NIU [81,82,83,84].

### 5.3. Special Considerations

Growing interest in the gut–eye axis has prompted investigation of microbiome-targeted therapies, including dietary modification, prebiotics, probiotics, synbiotics, and fecal microbiota transplantation (FMT), as potential adjunctive treatments for NIU. These approaches aim to restore microbial diversity, increase SCFA production, strengthen intestinal barrier integrity, and promote regulatory immune responses while suppressing pathogenic Th1/Th17 activity [15,16,19,70].

Among these, probiotics have received the greatest attention. Experimental studies suggest they may restore microbial homeostasis and promote immune tolerance, while FMT has been proposed to correct dysbiosis-driven immune dysfunction [15,70]. Supporting this concept, transplantation of gut microbiota from patients with Behçet disease or Vogt–Koyanagi–Harada disease into EAU models resulted in more severe ocular inflammation, suggesting that disease-associated microbial communities may directly influence uveitis severity [18,19,85].

Despite this compelling biological rationale, clinical evidence remains extremely limited. A systematic review identified only four small interventional reports evaluating probiotics or FMT in ocular disease, with heterogeneous conditions and generally low-level evidence; only one involved uveitis, and this was a single-patient case report [86]. Consequently, no microbiome-targeted intervention can currently be recommended for the routine prevention or treatment of NIU.

**Table 1:** Dietary interventions and their proposed effects on the gut-eye axis in non-infectious uveitis.

Intervention	Gut Microbiome Effects	Immunological Effects	Evidence in NIU
Mediterranean diet	Favorable modulation of microbial diversity and SCFA-producing taxa	↓ systemic inflammation, may promote regulatory immune responses	Indirect evidence [87,88]
Western diet	Dysbiosis, ↑ intestinal permeability	Pro-inflammatory immune activation	Indirect evidence [89,90,91]
Fermentable fiber	↑ butyrate and other SCFAs	↑ Tregs, ↓ Th1/Th17	EAU evidence [65,66]
Omega-3 fatty acids	Limited direct microbiome effects	↓ IFN-γ, ↓ IL-17, specialized pro-resolving mediators	EAU evidence [72]
Curcumin	May favor beneficial microbiota	NF-κB inhibition, ↓ IL-23/IL-17	Limited clinical evidence [77,78,79,80]
Probiotics	Modulation of microbial composition and restoration of microbial homeostasis	Potential promotion of regulatory immune responses	Preclinical evidence; insufficient clinical evidence [85]
Fecal microbiota transplantation	Restructuring of the intestinal microbial ecosystem	Broad effects on intestinal and systemic immune responses	Preclinical mechanistic evidence; no therapeutic clinical trials [92,93]

↑, increase; ↓, decrease.

Current evidence supports the biological plausibility of the gut-eye axis: experimental studies demonstrate that intestinal microorganisms, barrier integrity and microbial metabolites can modify ocular immune responses, while human studies have reported altered gut microbial profiles in several uveitis phenotypes. However, the human evidence is predominantly cross-sectional and heterogeneous, and is vulnerable to confounding by diet, geography, medication use, systemic disease and sequencing methodology. It remains uncertain whether dysbiosis is a cause or consequence of inflammation, which microbial changes are disease-specific, and whether microbiome-directed interventions improve NIU outcomes. Accordingly, the gut-eye axis remains a promising mechanistic and research framework rather than an established therapeutic target.

## 6. Sunlight, Vitamin D and Environmental Exposure

Vitamin D status and sunlight exposure may influence immune regulation through related but distinct pathways. Vitamin D has immunomodulatory actions beyond calcium homeostasis, whereas ultraviolet radiation produces both vitamin D-dependent and vitamin D-independent effects [94,95].

### 6.1. Biological Mechanisms

The active vitamin D metabolite calcitriol (1,25-(OH)_2_D_3_) regulates innate and adaptive immunity through the vitamin D receptor, which is expressed by numerous immune-cell populations [96]. Vitamin D receptor activation can promote tolerogenic immune responses by limiting dendritic-cell maturation and pro-inflammatory T-cell activity while supporting anti-inflammatory cytokine production and Treg-cell function [97,98,99,100,101,102]. Experimental vitamin D deficiency has also been associated with gut dysbiosis, disruption of intestinal and blood-retinal barriers, and increased retinal inflammatory signaling [103]. Ultraviolet radiation may exert additional vitamin D-dependent and independent immunological effects [95,104,105,106,107]; however, excessive ocular UVB exposure can disrupt the blood-aqueous barrier and induce anterior segment inflammation in experimental models [108]. These differing effects preclude treating sunlight exposure and vitamin D status and interchangeable.

### 6.2. Clinical Evidence

Human evidence concerning vitamin D and NIU can be divided into three distinct categories: observational associations with circulating vitamin D concentrations, genetic estimates of disease susceptibility, and clinical outcomes associated with supplementation.

Observational studies generally report lower serum 25-hydroxyvitamin D concentrations in patients with NIU than in healthy controls. A systematic review and meta-analysis involving more than 6000 participants found that vitamin D deficiency was associated with approximately two-fold greater odds of NIU (OR2.04) [100]. Lower circulating concentrations have also been associated with active disease or recurrence in several uveitis phenotypes, including HLA-B27-associated anterior uveitis, Behcet disease, Vogt-Koyanagi-Harada disease, sarcoidosis-associated uveitis, and JIA-associated uveitis [99,109,110,111]. A large healthcare database study similarly found lower odds of NIU among individuals with normal vitamin D concentrations [112]. However, these observational findings are susceptible to residual confounding, differences in the definition of deficiency, and reverse causation; for example, active disease may reduce sunlight exposure and alter health behaviors.

Mendelian randomization provides a complementary but distinct form of evidence. Genetically predicted reductions in circulating vitamin D were associated with increased susceptibility to uveitis and scleritis [113]. This approach reduced some sources of confounding and reverse causation, supporting a possible contribution of lower lifelong vitamin D status to disease susceptibility. However, genetic estimates do not establish that vitamin D supplementation prevents recurrence, induces remission, or treats established ocular inflammation.

Evidence concerning supplementation in established uveitis remains limited. Shih et al. retrospectively analyzed patients with non-infectious chronic anterior uveitis from the Systemic Immunosuppressive Therapy for Eye Diseases cohort [114]. The analysis compared 75 patients who received vitamin D supplementation for one year or less with 2688 patients who never received supplementation. At 16 months, the adjusted cumulative incidence of remission was 28.0% among vitamin D users and 13.5% among non-users. Vitamin D use was associated with a higher incidence of remission after adjustment for measured clinical characteristics (adjusted HR 2.43, 95% CI 1.36–4.33, *p* = 0.0027). Remission was defined as inactive disease for at least 90 days without anti-inflammatory treatment for ocular disease.

This finding is hypothesis-generating rather than evidence of treatment efficacy. Supplementation was not randomized, the indication for vitamin D use was unavailable, and baseline vitamin D concentrations were not reported; therefore, the supplemented patients were not established to be vitamin D deficient. The supplemented group was also small, doses ranged from 400 to 5000 units daily, and residual confounding and treatment-selection bias cannot be excluded. Prospective randomized trials are required to determine whether supplementation alters the course of established NIU. More broadly, evidence from other immune-mediated disease cannot be assumed to demonstrate an ocular anti-inflammatory benefit in NIU [115,116,117,118,119].

### 6.3. Special Considerations

Vitamin D assessment and supplementation should be considered according to the clinical indication. For general disease prevention, the 2024 Endocrine Society guideline advises against routine 25-hydroxyvitamin D testing in otherwise healthy adults without an established indication. Its recommendations primarily concern generally healthy populations and do not establish a specific approach for patients with NIU [120].

A separate indication applies to patients receiving prolonged systemic corticosteroid therapy, for whom fracture-risk assessment and appropriate calcium and vitamin D intake may form part of a broader bone-protection strategy. This indication concerns prevention of corticosteroid-associated skeletal complications rather than treatment of ocular inflammation [121].

Sarcoidosis requires caution because granulomatous production of 1,25-dihydroxyvitamin D can disturb calcium metabolism and vitamin D replacement may contribute to hypercalcemia. The American Thoracic Society recommends baseline serum calcium measurement in patients with sarcoidosis, including those without symptoms or signs of hypercalcemia. When vitamin D assessment is considered necessary and replacement is being contemplated, both 25-hydroxyvitamin D and 1,25-dihydroxyvitamin D should be measured [122].

At present, evidence is insufficient to recommend routine vitamin D testing or supplementation specifically to prevent NIU recurrence, induce remission, or reduce ocular inflammatory activity.

Latitudinal and seasonal variation have been described in several immune-mediated diseases, supporting interactions between environmental exposures and genetic susceptibility [123,124]. Similar patterns are increasingly recognized in uveitis, with large epidemiological studies demonstrating seasonal variation in disease incidence and activity associated with ambient temperature [125,126].

The strongest evidence exists for HLA-B27-associated anterior uveitis. In a New Zealand cohort, disease onset was highest during winter and independently associated with lower ambient temperature and higher humidity, while reduced sunlight hours were associated with disease onset on univariate analysis [127]. Earlier Australian data also demonstrated seasonal variation, although the pattern differed according to HLA-B27 status, suggesting that environmental influences vary between genetically susceptible populations [128].

The contribution of sunlight remains uncertain. Although sunlight promotes endogenous vitamin D synthesis and greater sun exposure has been associated with disease inactivity in vitamin D-deficient patients [109], ultraviolet radiation also exerts vitamin D-independent immunomodulatory effects, making it difficult to distinguish the relative contributions of sunlight, vitamin D status, and other seasonal exposures. Furthermore, epidemiological studies have not established a causal association between UV exposure and uveitis despite experimental evidence that direct ocular UV exposure can induce anterior segment inflammation [108,129].

Overall, these findings suggest that seasonal variation in NIU reflects the combined influence of vitamin D status, climate, environmental exposures, and host susceptibility rather than sunlight alone.

## 7. Obesity and Body Weight

Findings from experimental models and emerging clinical studies suggest that excess adiposity may also contribute to ocular inflammation [68,130,131].

### 7.1. Biological Mechanisms

Adipose tissue is an active endocrine organ, and excess adiposity produces a chronic low-grade inflammatory state through altered secretion of adipokines, cytokines and metabolic mediators [20,131,132,133,134]. Increased leptin, TNF-a and IL-6, together with reduced adiponectin, may favor inflammatory macrophage activity and impair metabolic and immune regulation [132,133,134]. Obesity and insulin resistance may thereby amplify the shared immunometabolic, vascular and barrier pathways outlined in Section 3 [68,132,135]. Body size may also influence the pharmacokinetics of fixed-dose biologic therapies, providing a possible mechanism through which adiposity could affect treatment exposure [136,137,138].

### 7.2. Clinical Evidence

Experimental studies demonstrate that obesity exacerbates ocular inflammation. In EAU, diet-induced obesity increases disease severity through enhanced Th1 responses, impaired regulatory immunity, and persistent inflammatory memory, while high-fat diets promote vitreous inflammation, oxidative stress, and increased pro-inflammatory cytokine expression, supporting a potential mechanistic role for metabolic dysfunction in ocular inflammation [68,139].

Clinical evidence linking obesity and metabolic dysfunction with NIU remains limited. Obesity is associated with several immune-mediated diseases, although comparable prospective evidence in NIU is lacking [131,140,141]. In a large retrospective primary-care cohort, poorer glycemic control was independently associated with recorded acute uveitis among patients with diabetes [142]. In a separate uncontrolled series of patients with poorly controlled diabetes and idiopathic anterior uveitis, persistently elevated HbA1c was associated with recurrence [143]. However, neither study establishes that improving glycemic control prevents uveitis or recurrence.

The emerging NIU evidence is observational. In a single-center cross-sectional study of 80 adults with NIU receiving adalimumab, higher BMI was associated with lower trough concentrations and poor clinical response, but not with anti-adalimumab antibody concentrations [138]. A separate cross-sectional study of 81 adults found anti-adalimumab antibodies in only five patients; these patients had a higher mean BMI and lower drug concentrations, although the small number of antibody-positive patients limits inference [136]. Neither study establishes that weight reduction improves treatment response.

In a retrospective single-center study of 40 children with JIA-associated uveitis, BMI was classified using age- and sex-specific percentiles and adalimumab was administered using weight-banded dosing. Higher BMI percentile was associated with lower trough concentrations, poorer response and anti-adalimumab antibodies [137]. However, growth, body size and the relationship between weight bands and drug exposure complicate interpretation; the findings should not be directly extrapolated to adult obesity management. Across these studies, adalimumab exposure may also be influenced by adherence, dosing interval, concomitant immunomodulation and inflammatory burden.

Evidence concerning BMI and other biologics is derived predominantly from non-ocular immune-mediated diseases [144,145]. It is therefore insufficient to recommend an alternative biologic for NIU solely based on BMI.

### 7.3. Special Considerations

Whether intentional weight loss improves uveitis outcomes or restores adalimumab response remains unknown. Weight management should therefore be recommended for established general and cardiometabolic indications rather than presented as a proven treatment for ocular inflammation.

Glucagon-like peptide-1 receptor agonists (GLP-1RAs) have emerged as a promising area of interest because they combine weight reduction with anti-inflammatory and immunometabolic effects. A large retrospective electronic health-record study found that GLP-1RA prescriptions were associated with a lower incidence of uveitis than matched non-use (RR 0.48; 95% CI 0.46–0.51), including in cohorts with and without diabetes [146]. GLP-1RA users had a higher incidence than SGLT2-inhibitor users in the primary analysis (RR 1.17; 95% CI 1.04–1.32); however, a sensitivity analysis restricted to monotherapy found no difference between these drug classes, while GLP-1RA monotherapy remained associated with lower incidence than metformin or insulin monotherapy. Exposure was defined from prescription records rather than confirmed use, and uveitis was identified using diagnostic codes. Residual confounding, including metabolic differences and prescribing patterns, remains possible, and findings in cohorts with and without diabetes do not establish independence from metabolic effects. These observational data concern incident uveitis and provide no evidence for treating established NIU.

At present, there is insufficient evidence to recommend weight-loss interventions or GLP-1RAs specifically to prevent or treat NIU. Nevertheless, optimization of metabolic health remains an important component of holistic care because of its established systemic benefits and possible influence on treatment exposure and response.

## 8. Physical Activity and Exercise

Regular exercise has demonstrated benefits across a range of autoimmune and chronic inflammatory diseases, and several biological mechanisms suggest potential relevance to NIU [147].

### 8.1. Biological Mechanisms

Beyond the shared pathways outlined in Section 3, contracting skeletal muscle acts as an endocrine organ by releasing myokines, particularly IL-6. Unlike chronically elevated IL-6 in inflammatory disease, exercise-induced IL-6 is released transiently and stimulates anti-inflammatory disease, exercise-induced IL-6 is released transiently and stimulates anti-inflammatory mediators, including IL-10 and IL1 receptor antagonist, while suppressing TNF-a [148,149,150,151]. Regular physical activity may also improve insulin sensitivity, reduce visceral adiposity, support neuroendocrine regulation and promote specialized pro-resolving mediators [149,151,152,153,154]. These effects vary with exercise intensity and duration: acute high-intensity exercise may transiently increase inflammatory mediators, whereas regular moderate-intensity activity is associated with lower systemic inflammatory biomarkers [148,155,156]. Whether these mechanisms influence NIU remains unproven. Proposed mechanisms by which physical activity could influence NIU are shown in Table 2.

### 8.2. Clinical Evidence

Direct clinical evidence evaluating physical activity in NIU is limited. One small observational study conducted a matched case–control study involving 39 patients with a recent recurrent acute anterior uveitis (RAAU) attack and 39 age-, sex-, and HLA-B27-matched patients with quiescent RAAU [157]. Mean self-reported exercise duration during the preceding month was 1.5 h per week in patients with a recent attack and 1.9 h per week in quiescent controls. Exercise duration was not significantly associated with a recent uveitis attack in univariate conditional logistic regression (OR per hour/week 0.86; 95% CI 0.65–1.13; *p* = 0.290. However, the small sample, retrospective self-reporting of exercise, limited assessment of exercise type and intensity, and imprecision of the estimate preclude conclusions regarding whether physical activity influences recurrence. No prospective or interventional studies have evaluated the effects of exercise on uveitis activity, recurrence, treatment response, corticosteroid requirements, or visual outcomes, and no disease-specific exercise recommendations have been developed.

In contrast, exercise is an established adjunctive therapy across multiple immune-mediated diseases that share pathogenic pathways with NIU. Randomized trials and meta-analyses demonstrate improvements in disease activity, physical function, fatigue, and quality of life in rheumatoid arthritis, axial spondyloarthritis, inflammatory bowel disease, multiple sclerosis, and psoriatic arthritis without increasing disease flares [54,147,158,159,160,161,162]. Accordingly, regular exercise is recommended in several international guidelines for these conditions [158,162].

Although these findings cannot establish an ocular benefit in NIU, they are clinically relevant because exercise modulates immune and metabolic pathways implicated in uveitis, and many patients with NIU have associated systemic inflammatory diseases for which physical activity forms part of multidisciplinary care [4,147].

### 8.3. Special Considerations

Regular physical activity may provide important psychosocial and general health benefits for patients with NIU. Chronic uveitis is frequently associated with fatigue, anxiety, depression, impaired health-related quality of life, and work disability, while visual impairment may reduce confidence, mobility, and participation in exercise [163].

Although exercise has not been specifically evaluated in NIU, evidence from rheumatoid arthritis, axial spondyloarthritis, inflammatory bowel disease, and multiple sclerosis consistently demonstrates improvements in fatigue, physical function, mood, and quality of life without increasing disease activity [158,162,164].

Exercise may also help mitigate the adverse effects of prolonged corticosteroid therapy by improving physical function, cardiovascular fitness, muscle health, and metabolic wellbeing, while potentially limiting weight gain and other treatment-related complications [165]. Improvements in metabolic health may also enhance biologic treatment response, although this remains untested in NIU [137,138,166]. Accordingly, appropriately tailored physical activity should be encouraged as a low-risk adjunct to standard care, even though there is currently insufficient evidence to recommend exercise specifically for the treatment of ocular inflammation.

**Table 2:** Proposed mechanisms by which regular physical activity may influence non-infectious uveitis.

Biological Pathway	Effects of Regular Physical Activity	Potential Relevance to NIU
Myokine signaling	Transient muscle-derived IL-6 stimulates IL-10 and IL-1 receptor antagonist and inhibits TNF-α signaling [167]	May reduce chronic systemic inflammation and promote immune homeostasis
Adaptive immune regulation	Modulation of Treg and effector T-cell responses [168]	May promote immune regulation and suppress pathogenic immune pathways implicated in NIU
Inflammation resolution	↑ specialized pro-resolving mediators (e.g., resolvin D1), enhanced macrophage phagocytosis/efferocytosis and neutrophil clearance [153]	Facilitates active resolution of inflammation rather than persistent immune activation
Metabolic health	Improved insulin sensitivity and body composition, reduced visceral adiposity, ↓ leptin, ↑ adiponectin [151,169]	Reduces metabolic drivers of chronic inflammation and may favorably influence treatment responsiveness
Gut–eye axis	Potential modulation of microbial diversity and SCFA-producing taxa, with effects on intestinal barrier function [170]	Potential reduction in dysbiosis-associated systemic immune activation
Neuroendocrine regulation	Modulation of autonomic nervous system and HPA-axis activity [171]	May reduce chronic stress-related immune activation
Systemic inflammation	Reduced circulating CRP, TNF-α and IL-6 with regular exercise training [155]	Lowers inflammatory burden associated with autoimmune disease
General health effects	Improved cardiovascular fitness, muscle strength, bone density, mood and fatigue [158,162,165]	May mitigate corticosteroid adverse effects and improve quality of life

Proposed mechanisms are based predominantly on evidence from exercise physiology, experimental models, and other immune-mediated disease; direct evidence demonstrating these pathways in NIU is currently limited. Abbreviations: ↑ = increase, ↓ = decreased, CRP = C-reactive protein; HPA = hypothalamic–pituitary–adrenal; IL = interleukin; NIU = non-infectious uveitis; SCFA = short-chain fatty acid; TNF = tumor necrosis factor; Treg = regulatory T cell.

## 9. Sleep

Sleep disturbance and circadian disruption have been implicated in several autoimmune diseases and may influence the development and clinical course of NIU through effects on inflammatory and neuroendocrine pathways [172].

### 9.1. Biological Mechanisms

Sleep regulates immune homeostasis through circadian, neuroendocrine and metabolic signaling [173]. Circadian clock genes influence immune-cell trafficking, cytokine production, cellular metabolism and inflammatory gene expression [24]. Sleep loss can disrupt these rhythms and activate systemic inflammatory pathways; acute sleep deprivation predominantly affects innate responses, whereas chronic restriction may promote sustained immune activation and impaired inflammatory resolution [173,174,175,176]. Sleep also interacts with the HPA, autonomic nervous system and melatonin signaling [173,177,178]. Melatonin has antioxidant and immunomodulatory effects within the eye and attenuates retinal inflammation in EAU, but there is no clinical evidence that melatonin supplementation improves NIU outcomes [178,179].

### 9.2. Clinical Evidence

Clinical evidence from immune-mediated diseases links sleep disturbance with increased disease activity, poorer quality of life, and adverse clinical outcomes. In rheumatoid arthritis, poor sleep correlates with greater disease activity and mediates approximately one-quarter of the relationship between disease activity and mental health-related quality of life [180,181]. Similarly, in inflammatory bowel disease, sleep disturbance is associated with active disease and predicts subsequent relapse, while comparable associations have been reported in systemic lupus erythematosus, psoriatic arthritis, and psoriasis [182,183,184,185,186]. These findings support a bidirectional relationship between sleep and immune-mediated inflammation.

Direct evidence in NIU remains limited. In a matched case–control study, Neti et al. compared 39 patients with a recent RAAU attack with 39 age-, sex-, and HLA-B27-matched patients with quiescent disease [157]. Patients with a recent attack reported shorted sleep duration than controls (6.3 ± 1.4 versus 7.4 ± 0.7 h per day), and sleeping fewer than seven hours per day was associated with a recent attack after adjustment for age, sex, HLA-B27 status, and concurrent systemic anti-inflammatory treatment (adjusted OR 12.12; 95% CI 1.37–107.17; *p* = 0.025). However, the very wide confidence interval indicates substantial imprecision. The small sample, retrospective reporting of sleep during the preceding month, possible recall bias, and case–control design preclude causal inference or interpretation of this estimate as an individual risk of recurrence. Exercise duration and alcohol consumption were also assessed in the same study but were not significantly associated with a recent attack [157].

Experimental studies further support this relationship. Sleep deprivation exacerbates EAU through activation of the IL-23/Th17/GM-CSF axis and increased retinal inflammation [187]. A systematic review of 15 predominantly cross-sectional studies found that patients with Behçet disease generally had poorer subjective or objective sleep measures than healthy controls [188]. However, these studies evaluated systemic Behçet cohorts rather than ocular disease specifically, and do not establish whether sleep disturbance affects uveitis activity or recurrence. In a separate cross-sectional study of 30 children with JIA, including only four with uveitis, the uveitis group reported more nocturnal awakenings, but the association was not statistically significant (*p* = 0.69) and sleep was not assessed using a validated sleep instrument [189].

No prospective studies have evaluated whether improving sleep quality reduces uveitis recurrence, and no interventional trials have examined sleep optimization as adjunctive therapy. Consequently, although inadequate sleep is a biologically plausible and potentially modifiable contributor to disease activity, current evidence remains insufficient to establish causality or support specific therapeutic recommendations.

### 9.3. Special Considerations

The relationship between sleep and NIU is likely bidirectional. Sleep disturbance may contribute to immune activation and disease recurrence, while recurrent ocular inflammation, visual impairment, treatment burden, and anxiety regarding visual outcomes may themselves impair sleep quality. Systemic corticosteroid therapy may further exacerbate insomnia and circadian disruption, potentially perpetuating this cycle [190,191].

Sleep disorders, particularly obstructive sleep apnea (OSA), are increasingly recognized as systemic inflammatory conditions characterized by intermittent hypoxia, oxidative stress, endothelial dysfunction, and elevated inflammatory cytokines [192]. Although OSA has been associated with several ocular diseases [193], its influence on the incidence, severity, or treatment response of NIU remains unknown.

Assessment of sleep quality may be considered as part of holistic uveitis care, particularly in patients with recurrent disease, chronic corticosteroid exposure, obesity, or associated systemic inflammatory disease. Although evidence is insufficient to recommend sleep interventions specifically to reduce uveitis activity, optimizing sleep hygiene and investigating suspected sleep disorders are likely to provide broader health and quality-of-life benefits.

## 10. Psychological Stress

Psychological stress may interact with inflammatory and neuroendocrine pathways relevant to NIU, although direct clinical evidence remains limited [194].

### 10.1. Biological Mechanisms

Psychological stress influences immune regulation principally through the HPA and sympathetic nervous system [22,26,30,195,196]. Physiological HPA-axis activation limits excessive inflammation through cortisol signaling. With prolonged stress, however, sustained neuroendocrine activation and reduced immune-cell sensitivity to glucocorticoids may permit persistent cytokine production and altered immune-cell trafficking [22,26,30,197,198,199]. Stress may also affect myelopoiesis and gut–brain-immune signaling, although these pathways overlap with the shared mechanisms described in Section 3 [30,199,200,201].

Importantly, stress-related immune effects are not uniformly pro-inflammatory and depend on the stressors, their duration and timing, and the phase of the immune response. In male Lewis rats with EAU, Song et al. applied chronic unpredictable mild stress from day 7 after immunization, during the disease effector phase [202]. Stressed animals developed milder ocular inflammation, with lower Th17-cell frequencies and IL-17 and IL-6 expression, and higher Treg-cell frequencies and IL-10, TGF-b and Foxp3 expression. The authors interpreted this as suppression of systemic Th17/Treg imbalance, potentially through HPA-axis-mediated immunosuppression. This experimental paradigm cannot be equated with perceived psychological stress in patients and does not establish that stress is clinically protective in NIU. Rather, it demonstrates that the effects of stress on ocular autoimmunity are context-dependent.

### 10.2. Clinical Evidence

Substantial evidence from immune-mediated diseases links psychological stress with increased disease activity and poorer clinical outcomes. In rheumatoid arthritis, perceived stress predicts subsequent increases in pain, fatigue, self-reported disease activity, and physical disability independent of medication use and disease duration [194,203]. Similarly, in inflammatory bowel disease, elevated stress precedes disease relapse, while comparable associations have been reported in systemic lupus erythematosus, multiple sclerosis, and psoriasis [204,205,206].

Evidence in uveitis is more limited but suggests that psychological distress is common. Patients with NIU report significantly higher perceived stress than healthy controls, particularly women, those with posterior or panuveitis, a history of depression, or receiving immunomodulatory therapy [207]. However, basal salivary cortisol concentrations did not differ from controls, suggesting that subjective psychological stress may not necessarily be accompanied by measurable alterations in HPA axis activity [207].

Whether psychological stress directly influences uveitis activity remains uncertain. Berlinberg et al. found no difference in perceived stress between active and inactive disease [207], whereas a German cohort reported higher stress levels in patients with active uveitis [208]. In the matched case–control study by Neti et al. an ST-5 stress score of at least three was associated with a recent RAAU attack after adjustment for age, sex, HLA-B27 status, and concurrent systemic anti-inflammatory treatment (adjusted OR 9.07; 95% CI 1.14–72.16, *p* = 0.037 [157]. Although this association is suggestive, the estimate was highly imprecise, as reflected by its wide confidence interval. Retrospective exposure reporting, the small sample of 39 matched pairs, and possible overlap between the stress and sleep measurements further limit causal interpretation and prevent the estimate from being applied as an individual recurrence risk. Separately, 57.9% of patients with HLA-B27-associated anterior uveitis in another study identified psychological distress as a perceived trigger for relapse [209].

Psychological comorbidity is also common in NIU, with anxiety, depression, and maladaptive coping associated with poorer vision-related quality of life [5,209]. Overall, current evidence suggests that psychological stress contributes substantially to patient wellbeing and may influence disease activity, although causality has yet to be established.

### 10.3. Special Considerations

The relationship between psychological stress and NIU is likely bidirectional. Chronic stress may contribute to immune activation and disease recurrence, while recurrent inflammation, visual impairment, treatment burden, and uncertainty regarding visual outcomes may themselves increase stress, anxiety, and depressive symptoms.

Assessment of psychological wellbeing may be incorporated into holistic uveitis care, particularly in patients with recurrent disease, posterior or panuveitis, prolonged immunosuppressive therapy, or significant vision loss. Screening for stress, anxiety, depression, sleep disturbance, and coping difficulties may identify patients who could benefit from psychological support alongside conventional ophthalmic management [210,211].

Mindfulness-based interventions, cognitive behavioral therapy, meditation, yoga, and structured stress-management programs have demonstrated improvements in psychological wellbeing, fatigue, and quality of life across several immune-mediate diseases, although effects on objective inflammatory disease activity remain inconsistent [212,213,214].

Direct interventional evidence is now available in NIU. Shantha et al. conducted a single-center, single-masked, waitlist-controlled randomized trial involving 100 adults with active or inactive NIU and at least mild symptoms of anxiety or depression [215]. Participants were randomized 1:1 to immediate access to a digital mindfulness program or a waitlist control. The intervention group was instructed to use the application for at least 10 min daily for eight weeks. At eight weeks, 70 participants completed the primary outcome assessment. After adjustment for baseline scores, the intervention was associated with lower anxiety scores than the control condition (mean difference in GAD-7, −1.70 points; 95% CI −3.17 to −0.23; *p* = 0.02). Depression and perceived stress scores also improved (PHQ-9 mean difference, −1.90; 95% CI −3.04 to −0.76; *p* = 0.001; PSS-10 mean difference, −3.69; 95% CI −6.00 to −1.37; *p* = 0.002). The between-group difference in vision related quality of life was not statistically significant (NEI VFQ-25 mean difference, 1.98; 95% CI −0.90 to 4.86; *p* = 0.18) [215].

These findings support digital mindfulness as a potential accessible adjunct for improving psychological wellbeing in selected patients with NIU. However, follow-up was limited to eight weeks, 30% of randomized participants did not complete the primary assessment, and the use of a waitlist rather than an active control may have increased expectancy or attention-related effects. Participants and study coordinators were not masked, and outcomes were self-reported. Although sensitivity analyses addressing missing data produced similar findings, longer-term effectiveness and generalizability remain uncertain. Importantly, the trial did not assess change in ocular inflammatory activity, recurrence, corticosteroid exposure, or immunosuppressive requirements. It therefore provides evidence of psychological benefit only and cannot determine whether mindfulness influences the clinical course of NIU.

## 11. Alcohol and Recreational Substances

Evidence linking alcohol or recreational substance use with NIU is limited and derives predominantly from experimental studies or other inflammatory diseases, with uncertain clinical applicability to uveitis.

### 11.1. Biological Mechanisms

The immunological effects of alcohol and recreational substances vary according to the substances, dose, duration and pattern of exposure.

Alcohol. Alcohol has dose- and context-dependent effects on immunity. Acute exposure may transiently suppress inflammatory responses, whereas chronic consumption can promote gut-derived endotoxaemia, Toll-like receptor signaling, oxidative stress and intestinal-barrier dysfunction [216,217,218,219,220].

Cannabis and cannabinoids. Cannabinoids may suppress inflammatory cytokine production and leukocyte recruitment through cannabinoid receptor 2 signaling [221,222]. Activation of the ocular endocannabinoid system reduces retinal inflammation and modulates T-cell responses in EAU, but these findings have not been demonstrated clinically [223,224,225,226].

Other recreational substances. Opioids, cocaine and psychostimulants have heterogeneous immunological effects, ranging from immunosuppression to innate immune activation, but evidence concerning autoimmune ocular inflammation is extremely limited [227,228].

These predominantly experimental mechanisms do not establish that alcohol or recreational substances influence NIU incidence, recurrence or treatment response.

### 11.2. Clinical Evidence

Clinical evidence linking alcohol consumption with NIU is limited to a small observational study. In the matched case–control study by Neti et al. alcohol consumption during the preceding month was reported by 6 of 39 patients with a recent RAAU attack and 3 of 39 patients with quiescent disease [157]. Alcohol consumption was not significantly associated with a recent attack in univariate conditional logistic regression (OR 2.50, 95% CI 0.49–12.89, *p* = 0.273). The wide confidence interval, small number of exposed participants, retrospective exposure reporting, and absence of detailed assessment of alcohol dose or drinking pattern mean that either a harmful or protective association cannot be excluded. No prospective or interventional studies have evaluated whether alcohol consumption influences uveitis incidence, recurrence, severity, treatment response, or visual outcomes.

A systematic review found no convincing evidence that moderate alcohol consumption substantially affects common ocular diseases, although uveitis was not specifically examined [229]. Evidence from other immune-mediated diseases is also inconclusive. Experimental studies and Mendelian randomization analyses have produced conflicting findings regarding autoimmune disease susceptibility. Any effects are likely to depend on dose, drinking pattern, and patient characteristics [230,231,232,233].

No epidemiological studies have evaluated whether recreational drug use influences uveitis incidence, recurrence, severity, or treatment response.

Clinical evidence for cannabinoids is equally limited. No studies have examined cannabis use in NIU. Although experimental models demonstrate anti-inflammatory effects, clinical studies in inflammatory bowel disease, multiple sclerosis, and rheumatoid arthritis suggest cannabinoids primarily improve symptoms rather than objective disease activity, and current guidelines do not recommend them as disease-modifying therapy [225,226,234,235].

There are similarly no clinical data evaluating opioids, cocaine, amphetamines, psychedelics, or other recreational substances in NIU [236,237].

Overall, the available evidence is insufficient to determine whether alcohol or recreational substance use influences the development, recurrence, or treatment response of NIU.

### 11.3. Special Considerations

In the absence of disease-specific evidence, patients with NIU should be counselled according to established public health recommendations regarding alcohol consumption and recreational substance use rather than presumed effects on ocular inflammation. Heavy alcohol consumption should be discouraged because of its established systemic health risks and biologically plausible pro-inflammatory effects.

Alcohol intake should be considered in patients receiving systemic immunomodulatory therapy, particularly methotrexate. In a large rheumatoid arthritis cohort, alcohol consumption below 14 UK units per week was not associated with increased hepatotoxicity, whereas intake exceeding 21 units per week significantly increased the risk of transaminitis [238]. Assessment should also consider underlying liver disease, obesity, diabetes, metabolic dysfunction, and concomitant hepatotoxic medications.

Cannabinoids should not be recommended as treatment for NIU outside clinical trials. Although experimental studies demonstrate anti-inflammatory effects, there is no clinical evidence that cannabis alters uveitis activity or reduces immunosuppressive requirements. Clinicians should instead remain alert to potential drug interactions, cognitive effects, impaired treatment adherence, and the broader health consequences of recreational substance use.

## 12. Patient Counselling and Clinical Practice

Lifestyle modification should be viewed as an adjunct to, rather than a replacement for, established pharmacological therapy in NIU. Its principal current contribution to personalized medicine is not the prescription of a uniform lifestyle program, but the integration of lifestyle assessment with the patient’s uveitis phenotype, systemic disease, treatment regimen, comorbidities and priorities.

Counselling should therefore be individualized. Smoking cessation should be routinely recommended, whereas dietary and physical-activity advice should primarily reflect established general-health guidance. Vitamin D testing and replacement should be based on recognized indications, corticosteroid-related bone-health requirements and disease-specific considerations, particularly altered calcium metabolism in sarcoidosis. In patients receiving adalimumab, body size may be considered alongside adherence, dosing interval, concomitant immunomodulation, inflammatory burden and anti-drug antibodies when investigating reduced drug exposure or response. Psychological assessment and support may be particularly relevant for patients experiencing anxiety, depression or stress related to NIU, although ocular disease-modifying benefits have not been demonstrated.

Alcohol and recreational substance use should be assessed in relation to medication safety, liver function, treatment adherence and systemic comorbidity rather than presumed effects on ocular inflammation. Weight-related discussion should similarly be conducted sensitively and without stigma, recognizing the biological, psychological, socioeconomic and treatment-related influences on body weight.

This approach uses lifestyle information to support shared decision-making, treatment safety and systemic health while avoiding unsupported claims that lifestyle modification prevents recurrence or treats established ocular inflammation. Table 3 summarizes the human NIU outcome supported for each factor and its appropriate clinical interpretation.

## 13. Limitations of Current Evidence

Current evidence linking lifestyle factors with NIU is derived predominantly from experimental models, observational studies, and extrapolation from other immune-mediated diseases. Although these studies provide important biological insights, they cannot establish causality or determine whether lifestyle modification directly influences disease activity, recurrence, treatment response, or visual outcomes.

Interpretation is further complicated by confounding, reverse causation, and the clustering of lifestyle behaviors with socioeconomic and environmental factors. Active disease, visual impairment, treatment-related adverse effects, and psychological distress may themselves alter lifestyle behaviors, making causal relationships difficult to establish. In addition, the marked heterogeneity of NIU, encompassing disorders with distinct immunopathogenic mechanisms and systemic associations, limits the generalizability of findings across disease subtypes.

The evidence base is further constrained by the absence of prospective longitudinal cohorts and adequately powered interventional trials, together with the confounding effects of corticosteroids, immunomodulatory therapies, and biologics. Standardized assessment of lifestyle exposures and validated biomarkers are also lacking. Consequently, the current literature should be regarded as hypothesis-generating rather than practice-changing. Future research should prioritize prospective cohorts, randomized lifestyle intervention trials, and integrated multi-omic approaches to identify patients most likely to benefit from personalized lifestyle modification alongside conventional therapy.

## 14. Future Directions

Since the earlier review of lifestyle factors in immune-mediated uveitis [14], the evidence base has expanded beyond its principal focus on smoking, diet, the gut microbiome and physical activity. Recent studies have examined adiposity in relation to adalimumab exposure and response, GLP-1 receptor agonists and incident uveitis, vitamin D supplementation, and psychological interventions in NIU. Although these findings remain preliminary and do not establish that lifestyle modification alters ocular inflammation, they permit a more clinically focused synthesis organized according to the outcomes studied, including disease incidence, inflammatory activity, recurrence, treatment response, psychological wellbeing and quality of life. This review therefore distinguishes NIU-specific clinical evidence from general health advice and from interventions that remain research hypotheses.

Advances in systems biology, including microbiome profiling, metabolomics, immunophenotyping, and digital health technologies, provide new opportunities to better understand how lifestyle factors influence immune regulation and ocular inflammation. Prospective registries integrating lifestyle measures with multimodal imaging, biomarkers, treatment response, and patient-reported outcomes may help identify modifiable risk factors and patients most likely to benefit from targeted interventions.

Priority areas for future research include Mediterranean-style dietary interventions, structured exercise programs, smoking cessation, vitamin D optimization, weight management, sleep optimization, psychological interventions, and microbiome-directed therapies. Clinical trials should evaluate not only disease activity and recurrence but also treatment response, visual outcomes, quality of life, and corticosteroid exposure.

Future studies should also investigate gene–environment interactions. In the UK Biobank, adherence to a healthy lifestyle attenuated genetic risk for diabetic retinopathy, suggesting that behavioral factors may modify inherited susceptibility to ocular disease [239]. Whether similar interactions exist in NIU remains unknown.

Ultimately, integrating clinical, genetic, immunological, metabolic, and behavioral data may support more personalized adjunctive counselling and the development of targeted interventions alongside conventional immunomodulatory therapy.

## 15. Conclusions

Lifestyle factors are increasingly recognized as important modulators of immune function and systemic inflammation, with emerging evidence suggesting they may influence the pathogenesis and clinical course of NIU. Although current evidence is derived largely from experimental models, observational studies, and related immune-mediated diseases, smoking, diet, physical activity, metabolic health, vitamin D status, sleep, and psychological wellbeing represent potentially modifiable factors relevant to ocular inflammation.

Lifestyle modification should be regarded as an adjunct to, rather than a replacement for, established immunomodulatory therapy. Smoking cessation has the strongest clinical evidence, while optimization of diet, physical activity, metabolic health, sleep, and psychological wellbeing represents a pragmatic, low-risk approach that supports both systemic health and holistic patient care.

Future prospective studies are required to determine whether targeted lifestyle interventions can improve disease activity, treatment response, visual outcomes, and quality of life. As the evidence base evolves, integrating lifestyle medicine with personalized immunomodulatory therapy may enable more precise, preventive, and patient-centered care for patients with NIU.

## Figures and Tables

**Table 3:** Human evidence and clinical interpretation of lifestyle factors in non-infectious uveitis.

Lifestyle Factor	Human NIU Evidence & Outcome	Suggested Advice
Smoking	Observational studies and meta-analysis associate smoking with uveitis susceptibility and, in some populations, greater inflammatory activity, complications or poorer outcomes [31,33,34,44,45,46,47,48,49,50,51]. No cessation study has demonstrated improved ocular outcomes.	Recommend cessation and provide support because of established general-health benefits. Explain that cessation has not been shown directly to modify the subsequent course of NIU.
Mediterranean-style diet	No study has directly evaluated a Mediterranean-style diet as an intervention for NIU; the rationale is derived from general-health and other immune-mediated disease evidence [69].	Encourage a balanced Mediterranean-style diet for general health, without presenting it as NIU treatment.
Vitamin D	Observational studies associate circulating vitamin D concentrations with NIU susceptibility or activity, while Mendelian randomization estimates concern susceptibility [99,100,109,110,111,112,113]. One retrospective cohort associated supplementation with greater remission of chronic anterior uveitis, but no randomized ocular trial had demonstrated efficacy [114].	Do not test or supplement solely because of NIU. Follow general prevention and corticosteroid-related bone-health guidance. In sarcoidosis, obtain baseline serum calcium and, if vitamin D replacement is being considered, assess both 25-hydroxyvitamin D and 1,25-dihydroxyvitamin D.
Omega-3 intake	Evidence in uveitis is experimental [72,73]; no human study has demonstrated that omega-3 supplementation improves NIU outcomes	Encourage dietary sources as part of a balanced diet; do not recommend supplements specifically for NIU.
Weight and metabolic health	Higher BMI has been associated with lower adalimumab exposure or poorer response in adults and pediatric NIU, although findings concerning anti-drug antibodies are inconsistent [136,137,138]. Poor glycemic control has been associated with uveitis occurrence or recurrence in patients with diabetes [142,143]. GLP-1RA prescription studies concern incident uveitis and are comparator-dependent [146]. No study has shown that intentional weight loss improves NIU outcomes.	Support weight and metabolic health for established indications and corticosteroid-risk reduction, without promising improved ocular control.
Physical activity	One small matched case–control study found no significant association between self-reported exercise duration and a recent RAAU attack (OR per hour/week 0.86; 95% CI 0.65–1.13) [157]. No interventional study has evaluated ocular outcomes.	Encourage appropriate aerobic and strength activity for general health, not as established NIU therapy.
Sleep	One small matched case–control study associated sleeping fewer than seven hours daily with a recent RAAU attack, but the estimate was imprecise (adjusted OR 12.12; 95% CI 1.37–107.7) [157]. No study has shown that improving sleep prevents recurrence.	Assess clinically relevant sleep problems and encourage healthy sleep practices for general health.
Psychological wellbeing	Observational findings concerning perceived stress and uveitis activity are mixed [157,207,208,209]. An eight-week randomized trial found improvements in anxiety, depression and perceived stress with digital mindfulness, but not vision-related quality of life; ocular disease activity and treatment outcomes were not assessed [215].	Assess psychological distress and offer appropriate psychological support. Digital mindfulness may improve psychological wellbeing but should not be presented as treatment for ocular inflammation.
Alcohol	One small matched case–control study found no significant association between alcohol consumption and a recent RAAU attack [157]. Effects on other NIU outcomes remain unknown.	Follow public-health advice and consider medication and liver safety; do not imply an ocular benefit from reduction.
Recreational substances	No human study has demonstrated that cannabis or other recreational substances modify NIU incidence, activity, recurrence or treatment response.	Do not recommend as NIU treatment; assess use where relevant to safety, interactions, cognition, or adherence.

Lifestyle measures are adjuncts to, not substitutes for established NIU treatment. General-health advice does not imply a demonstrated ocular disease-modifying effect. NIU, non-infectious uveitis; RAAU, recurrent acute anterior uveitis.

## Data Availability

No new data were created or analyzed in this study. Data sharing is not applicable to this article.

## Supplementary Materials

The following supporting information can be downloaded at https://www.mdpi.com/article/10.3390/jpm16100511/s1. Table S1: Searches; Table S2: Key Human Studies of Non-Infectious Uveitis.
