# Supplementary material for: Lifestyle Modifiers of Non-Infectious Uveitis: A Narrative Review of Current Evidence and Future Directions
Source: J Pers Med. 2026 Sep 30;16(10):511. doi: 10.3390/jpm16100511 (PMC13706412; doi:10.3390/jpm16100511)
Supplement: Supplementary file 1 [file jpm-16-00511-s001.zip › jpm-4558316-supplementary table S1.pdf]

Supplementary Table S1: Searches

Database	Search Strategy	Dates searched; results retrieved
**MEDLINE via Ovid**	1. exp uveitis/<br>2. uveit*.ti,ab,kf.<br>3. 1 or 2<br>4. (smok* or tobacco or vap* or diet* or nutrition* or mediterranean or curcumin or omega-3 or obes* or adiposity or body mass index or exercis* or physical activ* or sleep* or circadian or stress* or mindfulness or alcohol* or cannabis or cannabinoid* or microbiom* or probiotic* or vitamin D or sunlight).ti,ab,kf.<br>5. 3 and 4	27 September 2026;<br>1613 records
**PubMed**	1. Uveitis[MeSH Terms]<br>2. uveit*[tiab]<br>3. #1 OR #2<br>4. (smok*[tiab]<br>5. tobacco[tiab]<br>6. vap*[tiab]<br>7. diet*[tiab]<br>8. nutrition*[tiab]<br>9. mediterranean[tiab]<br>10. curcumin[tiab]<br>11. omega-3[tiab]<br>12. obes*[tiab]<br>13. adiposity[tiab]<br>14. "body mass index"[tiab]<br>15. exercis*[tiab]<br>16. "physical activity"[tiab]<br>17. sleep*[tiab]<br>18. circadian[tiab]<br>19. stress*[tiab]<br>20. mindfulness[tiab]<br>21. alcohol*[tiab]<br>22. cannabis[tiab]<br>23. cannabinoid*[tiab]<br>24. microbiom*[tiab]<br>25. probiotic*[tiab]	27 September 2026;<br>1,868 hits summed across individual searches (overlap possible)

	26. "vitamin D"[tiab]<br>27. sunlight[tiab])<br>28. 3 AND 4 (197 records)<br>29. 3 AND 5 (20 records)<br>30. 3 AND 6 (2 records)<br>31. 3 AND 7 (137 records)<br>32. 3 AND 8 (54 records)<br>33. 3 AND 9 (292 records)<br>34. 3 AND 10 (34 records)<br>35. 3 AND 11 (5 records)<br>36. 3 AND 12 (96 records)<br>37. 3 AND 13 (1 record)<br>38. 3 AND 14 (68 records)<br>39. 3 AND 15 (84 records)<br>40. 3 AND 16 (17 records)<br>41. 3 AND 17 (53 records)<br>42. 3 AND 18 (18 records)<br>43. 3 AND 19 (440 records)<br>44. 3 AND 20 (1 record)<br>45. 3 AND 21 (68 records)<br>46. 3 AND 22 (5 records)<br>47. 3 AND 23 (18 records)<br>48. 3 AND 24 (139 records)<br>49. 3 AND 25 (31 records)<br>50. 3 AND 26(76 records)<br>51. 3 AND 27(12 records)	
**Embase via Ovid**	1. exp uveitis/<br>2. uveit*.ti,ab,kf.<br>3. 1 or 2<br>4. (smok* or tobacco or vap* or diet* or nutrition* or mediterranean or curcumin or omega-3 or obes* or adiposity or body mass index or exercis* or physical activ* or sleep* or circadian or stress* or mindfulness or alcohol* or cannabis or cannabinoid* or microbiom* or probiotic* or vitamin D or sunlight).ti,ab,kf.<br>5. 3 and 4	27 September 2026;<br>2107 records

**Cochrane Database of Systematic Reviews**	1. Uveitis<br>2. Smoking<br>3. Tobacco<br>4. Vaping<br>5. Diet<br>6. Nutrition<br>7. Adiposity<br>8. Body Mass Index<br>9. Exercise<br>10. Physical Activity<br>11. Sleep<br>12. Stress<br>13. Cannabis<br>14. Cannabinoids<br>15. Probiotics<br>16. Vitamin D<br>17. Sunlight<br>18. 1 AND 2 (7 records)<br>19. 1 AND 3 (4 records)<br>20. 1 AND 4 (0 records)<br>21. 1 AND 5 (9 records)<br>22. 1 AND 6 (9 records)<br>23. 1 AND 7 (0 records)<br>24. 1 AND 8 (22 records)<br>25. 1 AND 9 (15 records)<br>26. 1 AND 10 (55 records)<br>27. 1 AND 11 (14 records)<br>28. 1 AND 12 (25 records)<br>29. 1 AND 13 (0 records)<br>30. 1 AND 14 (0 records)<br>31. 1 AND 15 (4 records)<br>32. 1 AND 16 (0 records)<br>33. 1 AND 17 (4 records)	27 September 2026;<br>168 hits summed across individual searches (overlap possible)

† Searches were conducted without a publication-year restriction. English-language and publication-type eligibility criteria were applied during selection. The original search strings were not retained; this table reports the documented updated searches.
