# Supplementary material for: Lifestyle Modifiers of Non-Infectious Uveitis: A Narrative Review of Current Evidence and Future Directions
Source: J Pers Med. 2026 Sep 30;16(10):511. doi: 10.3390/jpm16100511 (PMC13706412; doi:10.3390/jpm16100511)
Supplement: Supplementary file 1 [file jpm-16-00511-s001.zip › jpm-4558316-supplementary table S2.pdf]

Supplementary Table S2: Key Human Studies of Non-Infectious Uveitis

Lifestyle Factor	Study design	Population and uveitis phenotype (n)	Exposure/intervention	Comparator	Outcome and follow-up	Effect estimate (95% CI)	Principal limitations
**Smoking & tobacco exposure**	Kuč et al. 2026 [44]; systematic review and meta-analysis of seven observational studies: four retrospective case-control, one retrospective cohort and two cross-sectional studies	Adults with ophthalmologist-confirmed uveitis and no systemic inflammatory disease. Phenotypes included anterior, intermediate, posterior and panuveitis, plus presumed ocular histoplasmosis syndrome. Primary cigarette-smoking meta-analysis: 1,557 cases and 4,293 controls	Current and former cigarette smoking, as defined in each study; exposure definitions varied	Participants without uveitis, including healthy and other ophthalmic controls; reference smoking categories varied	Association between smoking and presence of uveitis	**Current smoking:** pooled unadjusted OR 1.97 (95% CI 1.71-2.27, I^2=46%; four retrospective studies)<br><br>**Former smoking:** pooled unadjusted OR 1.23 (95% CI 1.05-1.44; I^2=79%; three retrospective studies)	Small number of observational studies; pooled estimates not uniformly adjusted for confounders; substantial heterogeneity for former smoking; inconsistent definitions of smoking intensity, duration and cessation; heterogeneous uveitis phenotypes and controls; inclusion of POHS; exclusion of systemic inflammatory diseases limits generalizability; no evidence that smoking cessation changes ocular outcomes
	Lin et al.2010 [33]; single-centre retrospective case-control study	564 patients with ocular inflammation and 564 comprehensive eye-clinic controls. Cases included anterior uveitis (n=331),	Self-reported current, past or never smoking. Current and past smoking were combined as “ever smoking” for phenotype analyses	Comprehensive eye-clinic patients without current or previous ocular inflammation; never smoking	Association between smoking and the presence and anatomical phenotype of ocular inflammation	Adjusted for age, sex, race and area-level median income. **Ever smoking and all ocular inflammation:** OR 2.2 (95% CI 1.7-3.0,	Retrospective, single-centre design; mixed infectious and non-infectious population; some patients had scleritis or episcleritis; smoking was self-

		intermediate uveitis (n=57), posterior uveitis (n=25) and panuveitis (n=133); 31 had inflammation at more than one anatomical location. The population included 515 patients with non-infectious or idiopathic inflammation and 49 with infectious uveitis		was the exposure reference		p<0.001). **Non-infectious or idiopathic inflammation:** OR 2.1 (95% CI 1.6-2.8, p<0.001). **Anterior uveitis:** OR 1.7 (95% CI 1.2-2.4, p=0.002); **intermediate uveitis:** OR 2.7 (95% CI 1.4-5.6, p=0.005); **posterior uveitis:** OR 3.2 (95% CI 1.3-7.9, p=0.014); **panuveitis:** OR 3.9 (95% CI 2.4-6.1, p<0.001)	reported; no information on pack-year, exposure timing, tobacco type or passive smoking; incomplete smoking questionnaires, particularly among cases; residual confounding and selection bias; temporality and causality cannot be established
	Lin et al.2010 [33]; phenotype subgroup analysis from the same retrospective case-control study	Intermediate uveitis with CME (n=18) or without CME (n=39); panuveitis with CME (n=31) or without CME (n=102). Phenotype groups included infectious and non-infectious uveitis	Self-reported ever smoking	The same 564 comprehensive eye-clinic controls without ocular inflammation – not patients with uveitis without CME	Association between smoking and uveitis phenotypes accompanied by CME	Adjusted for age, sex, race and area-level median income. **Intermediate uveitis with CME:** OR 8.4 (95% CI 2.5-28.8, p=0.001); **intermediate uveitis without CME:** OR 1.5 (95% CI 0.6-3.8, p=0.342). **Panuveitis with CME:** OR 8.0 (95% CI 3.3-19.5, p<0.001); **panuveitis without CME:** OR 3.1 (95% CI 1.8-5.2, p<0.001)	Small phenotype subgroups and wide confidence intervals; comparator was an eye-clinic control group rather than patients with uveitis without CME; cross-sectional phenotype comparison cannot show that smoking preceded or caused CME; mixed infectious and non-infectious etiologies; same exposure-measurement and residual-confounding limitation as the principal analysis
	Roesel et al.2011 [34]; single-centre	350 adults with NIU; anterior (n=151), intermediate (n=79),	Self-reported current smoking; smoking intensity assessed using	Non-smokers within the NIU cohort	Uveitis activity at cross-sectional assessment; topical	**Active uveitis:** adjusted OR 1.7 (95% CI 1.1-2.7;	Cross-sectional, single-centre design; cannot establish

	observational cross-sectional study	posterior (n=49) and panuveitis (n=71); 136 had an associated immune-mediated disease. Current smokers: n=115 (32.9%)	cigarettes/day and pack-years		and systemic corticosteroid use; second-line immunosuppression; prevalent macular edema, cataract and other complications. No longitudinal follow-up	p=0.023); covariates in the multivariable model were not specified. In anterior uveitis, smokers more frequently had anterior chamber cell (39% vs 21%; p=0.008) and used topical corticosteroids (44.8% vs 34.8%; p=0.002). There was no difference in second-line immunosuppression (p=0.642) or number of immunosuppressive agents (p=0.794). Greater pack-years, but not smoking status alone, were associated with prevalent macular edema (p=0.020) and cataract (p=0.222)	temporality or causality; no non-uveitis control group; smoking was self-reported and the handling of former smokers was unclear; activity, treatment use and complications were assessed at one time point; multivariable covariates were not reported; possible confounding by disease duration; pack-year effect estimates inadequately reported and rounded; no assessment of whether cessation modifies outcomes
	Thorne et al.2008 [45], single-centre retrospective cross-sectional study	208 patients with non-infectious intermediate uveitis involving 363 eyes; 89 patients (43%) had CME in at least one eye at presentation. Smoking status was available for 96%; current smokers	Self-reported current, former and never smoking at presentation; cigarettes/day, duration and pack-years were recorded for smokers	Never smokers with intermediate uveitis	Presence of CME in either eye at first presentation to a tertiary uveitis centre; no longitudinal follow-up	Adjusted for age, sex, race, duration of uveitis, topical and systemic corticosteroid therapy, diabetes, hypertension, active intraocular inflammation and epiretinal membrane. **Current**	Retrospective cross-sectional design; outcome was prevalent CME at tertiary-centre presentation rather than incident CME; possible referral and treatment-by-indication bias; smoking was self-

		n=63, former smokers n=30, never smokers approximately n=106				**versus never smoking:** OR 3.90 (95% CI 1.43-10.66, p=0.008). **Former versus never smoking:** OR 1.97 (95% CI 0.99-3.94, p=0.055). Among current smokers, each additional cigarette/day was associated with OR 1.04 (95% CI 1.01-1.7, p=0.005) for prevalent CME	reported; timing of smoking and cessation relative to CME was unavailable; relatively small smoking subgroup and imprecise estimates; CME was clinically diagnosed with angiographic or OCT confirmation only when available; residual confounding remains possible; no evidence that cessation reduces subsequent CME risk
	Galor et al.2010 [31]; multicentre retrospective longitudinal SITE cohort	2,676 patients with active non-infectious ocular inflammation; anterior, intermediate, posterior or panuveitis, scleritis, mucous membrane pemphigoid and other ocular inflammatory disorder	Smoking status recorded from clinical records as current, former or never smoking; quantity and duration were not assessed	Never smokers; former smokers examined separately	Time from active inflammation to quiescence and from quiescence to recurrence; variable longitudinal follow-up. Median time to recurrence was 7.8 months in current smokers, 9.4 months in never smokers and 10.7 months in former smokers	Current versus never smokers: recurrence aHR 1.19 (95% CI 1.03-1.37). No significant difference in time to quiescence: never versus current smoking aHR 1.09 (95% CI 0.98-1.22). Models included relevant demographic and disease characteristics and center effects	Retrospective smoking ascertainment; no information on dose, duration or changes in smoking status; 1,021 otherwise eligible patients lacked smoking data; heterogeneous population not restricted to uveitis; possible residual confounding by treatment adherence, healthcare utilisation and comorbidity. Observational associations do not demonstrate that cessation alters ocular outcomes

	Arriola-Villalobos et al.2018 [48]; single-centre cross-sectional study	156 adults with NIU attending a tertiary clinic in Madrid: anterior uveitis 75, intermediate uveitis 26, posterior uveitis 21 and panuveitis 34	Smoking status classified as active (n=40), former (n=47) or never smoking (n=69); smoking intensity and cumulative exposure were not reported	Never smokers	Vision-related quality of life measured using the NEI VFQ-25 composite score at a single assessment	Active versus never smoking: ES 0.81 (95% CI 0.51-1.29, p=0.34). Smoking was not included in the multivariable model because it was not associated with the outcome in the bivariate analysis	Cross-sectional design precludes temporal inference; single tertiary-centre population; smoking exposure was incompletely characterised; analysis was underpowered to exclude a clinically relevant association; no independently adjusted smoking estimate; heterogeneous NIU phenotypes and potential residual confounding
	Helm and Holland. 1995 [47]; retrospective consecutive case series	20 patients with idiopathic intermediate uveitis treated with posterior sub-Tenon triamcinolone; 18 were eligible for visual-response analysis	History of smoking obtained from a baseline questionnaire; current/former status, dose and duration were not reported	Patients without a smoking history	Improvements in ≥2 Snellen lines following the initial injection; median overall follow-up 23.5 months (range 1-50). Twelve of 18 patients responded	Smoking history was present in 5/6 nonresponders versus 2/11 responders with known smoking status (p=0.03). After excluding children aged <15 years, the difference was no longer statistically significant: 5/6 versus 2/7 (p=0.073). No adjusted effect estimate or CI was reported	Very small retrospective, uncontrolled series; smoking status unavailable for one responder and poorly characterised; age confounded the association, with no multivariable adjustment; unmasked, non-standardised visual-acuity assessment; variable concomitant topical or systemic treatment; visual response could reflect macular edema, cataract or

							natural disease fluctuation
	Kempen et al. 2016 [49]; multicentre retrospective STIE cohort	510 patients comprising 849 eyes with primary non-infectious intermediate uveitis, including pars planitis and cases associated with systemic inflammatory disease	Smoking status obtained from clinical records and classified as current, former, never or unknown; intensity, duration and changes during follow-up were not reported	Never smokers	Remission, defined as absence of inflammatory activity at ≥2 visits spanning ≥90 days without ocular or systemic anti-inflammatory treatment; 1,934 eye-years of follow-up. Relapse following remission was also examined	Remission: current versus never smoking, aHR 0.79 (0.46-1.35); former versus never smoking, aHR 0.97 (0.54-1.76). Relapse: current smokers versus nonsmokers, aHR 2.08 (0.80-4.08). Neither association was statistically significant	Retrospective tertiary-centre cohort; smoking status unknown for 138/849 eyes and exposure incompletely characterised; potential residual confounding and selection bias; remission depended partly on treatment withdrawal and clinical management; relapse analysis included only 37 events and had limited precision; observational findings do not evaluate smoking cessation
	Levin et al.2014 [50]; multicentre retrospective SITE cohort	1,077 patients comprising 1,510 eyes with non-infectious uveitis and visual acuity <20/40 principally attributed to macular edema; anterior, intermediate, posterior and panuveitis included	Smoking status obtained from clinical records and classified as current, former, never or unknown; smoking intensity and cumulative exposure were not reported	Never smokers	Time to improvement of ≥2 lines of visual acuity after established visually significant uveitic macular edema; 2,060 eye-years of observation with median eye follow-up of 112 days. Six-months improvement: 52.7% in current, 50.3% in former and 54.2% in never smokers	Current versus never smoking: aHR 0.97 (95% CI 0.83-1.13); former versus never smoking: aHR 1.02 (95% CI 0.84-1.24). Neither association was statistically significant	Retrospective tertiary-centre cohort; smoking exposure incompletely characterised; heterogenous uveitis phenotype and treatments; macular edema diagnosed clinically in 77% of eyes rather than by OCT or angiography; visual improvement used as a surrogate

							for improvement in edema; incomplete follow-up and potential referral bias; residual confounding remains possible
	Brodie et al.2024 [51]; single-centre retrospective cohort	2,092 patients comprising 2,763 eyes with acute anterior uveitis; included idiopathic and immune-mediated NIU as well as viral and other infectious uveitis; chronic anterior uveitis was excluded	Smoking status recorded at initial presentation as current, ex-smoker or nonsmoker; ex-smokers were defined as smoke-free for ≥28 days	Nonsmokers	Ipsilateral recurrence and subsequent contralateral eye involvement; median follow-up 8.9 years, totalling 19,794.9 eye-years	Ipsilateral recurrence: current smokers aHR 0.932, p=0.736; ex-smokers aHR 1.004, p=0.853. Contralateral involvement: current smoking aHR 0.848, p=0.426; ex-smokers aHR 1.370, p-0.055. Confidence intervals were not reported	Retrospective study; smoking assessed only at presentation and may have changed during prolonged follow-up; no pack-year, intensity or duration data; mixed infectious and non-infectious population; an ex-smoker threshold of only 28 days does not represent sustained cessation; possible referral and residual confounding; no evaluation of a cessation intervention
	Hsu et al.2025 [53].; multi-institutional retrospective propensity-matched TriNetX cohort	419,325 adults coded as e-cigarette users and 419,325 matched non-users in US electronic health records; patients with recorded traditional cigarette use and selected uveitis-associated	At least two records containing ICD-10-CM codes for vaping-related disorder (U07.0) or unspecified nicotine dependence (F17.20) between September 2019 and December 2023	Propensity-matched patients without the exposure codes	First recorded diagnosis of uveitis, including iridocyclitis, choroidal inflammation, posterior cyclitis, retinal vasculitis and panuveitis; follow-up examined from 7 days to 4 years	Any incident uveitis: HR 2.53 (95% CI 2.33-2.76). Increased associations were reported throughout follow-up, from HR 6.35 (95% CI 3.54-11.37) at 7 days to HR 2.58 at 4 years (95% CI 2.37-2.81)	Retrospective diagnosis codes may identify vaping-related illness or unspecified nicotine dependence rather than habitual e-cigarette use; no information on frequency, duration, device or dose; uveitis was code-

		comorbidities were excluded					defined without phenotype or etiological validation and was not restricted to NIU; possible exposure and outcome misclassification, surveillance bias and residual confounding; the strong association within 7 days raised concern about ascertainment or reverse-temporality effects; cannot establish causality
	Malek Mahdavi et al.2019 [61]; case-control study with propensity-score matching	192 Azari patients with Behcet disease, 822 unaffected siblings and 373 unrelated healthy controls; 99/192 Behcet patients had uveitis. Propensity-matched analysis included 147 patients and 294 participants in each control group	Self-reported smoking before the first Behcet symptom. Ever smoking was defined as ≥100 lifetime cigarettes; current and former smokers were combined. Duration, cigarettes/day and pack-years were recorded	Never smokers	Primary outcome: presence of Behcet disease. Secondary analysis among Behcet patients examined the presence of uveitis and baseline ocular inflammatory activity using the Total Inflammatory Activity Index; cross-sectional assessment without longitudinal follow-up	Behcet-associated uveitis: ever versus never smoking, unadjusted OR 1.313 (0.718-2.402, p=0.232). Baseline ocular activity did not differ significantly (mean TIAI 4.11 vs 3.82, p=0.427). After propensity matching, ever smoking was not associated with Behcet disease compared with unaffected siblings, aOR 1.39 (0.80-2.41), or unrelated controls, aOR 1.45 (0.82-2.59); these	Single-centre, ethnically restricted population; smoking and historical disease activity were self-reported, created recall bias; only 51 patients were ever smokers and only three female patients smoked; uveitis was treated as a binary manifestation without anatomical phenotype or longitudinal activity outcomes; the uveitis analysis was not adjusted for confounders; no

						adjusted estimates concerned Behcet susceptibility, not uveitis	assessment of cessation effects
	Tuna et al. 2015 [62]; retrospective medical-record study	209 adults with Behcet disease in Turkey; 73 (34.9%) had ocular involvement	Current or former smoking combined (n=103); smoking history obtained from medical records	Never smoking (n=106)	Prevalent ocular involvement; no longitudinal follow-up	Ocular involvement: 41.7% in ever-smokers vs 28.3% in never-smokers (unadjusted p=0.042). In logistic regression, smoking alone was not significantly associated with ocular involvement (p=0.153); the combined smoking/HLA-B51 term was associated with ocular involvement (OR 2.19, 95% CI 1.09-4.41, p=0.028)	Retrospective single-centre study; ocular phenotype and activity were not characterised; current and former smokers were combined; temporal relationship could not be established; regression considered smoking and HLA-B51 but did not comprehensively adjust for age, sex, treatment or other confounders; interaction modelling was incompletely described
**Diet & Nutrition**	Kalyana Chakravarthy et al. 2018 [63]; cross-sectional case-control microbiome study	13 Indian adults with NIU: idiopathic uveitis (n=10) or VKH (n=3); anatomic phenotypes were not reported	Gut bacterial composition measured from a single stool sample using 16S rRNA sequencing	13 healthy controls matched for age, sex, ethnicity, geographic origin and reported diet	Microbial diversity and differential taxonomic abundance; single-time-point assessment	Uveitis and control microbiomes formed substantially different clusters. Of 204 differentially abundant operational taxonomic units, 180 were enriched in controls and 24 in patients with uveitis (Benjamini-Hochberg-adjustment p<0.05). Adjusted clinical	Very small, cross-sectional study; heterogeneous and incompletely characterised uveitis; only three patients had a defined autoimmune phenotype; medication and disease-activity effects were not adequately assessed; dietary matching was reported but dietary exposure was not

						effect estimates and Cis were not reported	quantified; multiple microbiome comparisons; associations cannot establish whether dysbiosis preceded or resulted from uveitis
	Allegri et al.2010 [78]; uncontrolled prospective before-after study	122 adults enrolled with recurrent anterior uveitis; 106 completed follow-up. Etiologies included autoimmune disease (n=56), herpetic uveitis (n=28), and other or unknown causes (n=22, including toxoplasmosis, tuberculosis, and Lyme disease)	Oral phosphatidylcholine-curcumin complex (Meriva; Norflo), 600mg twice daily (1,200 mg/day), added to existing therapy	Each patient's relapse history during the preceding year; no concurrent placebo or untreated control group	Number and severity of relapses, symptoms and tolerability over 12-18 months	Total relapses decreased from 275 in the year before treatment to 36 during treatment (p<0.001); patients experiencing relapse decreased from 106 to 19. No adjusted estimate or CI was reported	No randomisation, blinding or concurrent control; mixed infectious and non-infectious etiologies, concomitant topical and systemic corticosteroids, immunosuppressants and antimicrobial treatments were continued; potential regression to the mean and temporal changes in disease; 16 participants did not complete the study; relapse outcomes were not independently masked; no adjusted analysis
	Allegri et al.2022 [80]; single-centre prospective observational comparative study	26 adults with bilateral, recent-onset non-infectious uveitic macular edema; 43 eyes analysed. Phenotypes included intermediate, posterior and	Curcumin with polyvinylpyrrolidone hydrophilic carrier (CurcuWIN), 60mg per capsule twice daily for 12 months; administered with prednisone for the first 6 months and then continued alone	Standard prednisone treatment alone: initially 1 mg/kg/day, maximum 50 mg/day, followed by a specific taper	BCVA and central macular thickness at 3,6 and 12 months; secondary outcomes included FAZ, IOP and safety	At 12 months, mean BCVA was better with curcumin than with standard therapy alone (0.19±0.02 vs 0.40±0.07 logMAR; p≤0.01). In the curcumin group,	Very small, non-randomised, unmasked, single-centre study; treatment assigned according to physician practice; heterogeneous phenotypes and

		panuveitis associated with idiopathic uveitis, sarcoidosis, Behcet disease, multiple sclerosis or autoimmune retinal vasculitis		(11 patients; 18 analysed eyes)		mean CMT decreased from 320.0±40.8µm at baseline to 272.0±51.9µm at 12 months (p≤0.01). CIs and adjusted between-group estimates were not reported	baseline imbalance, including more sarcoidosis in control group; analysis included both eyes of some patients; no placebo; possible residual confounding; manufacturer supported editorial and graphical assistance
	Lal et al.;1999 [77]; uncontrolled, open-label clinical study	53 patients with chronic anterior uveitis enrolled; 32 completed treatment. Fifteen had recurrent disease; 14 completers with strong PPD responses also received antituberculous therapy	Unformulated curcumin of >95% purity, 375mg oral three times daily (1,125 mg/day) for 12 weeks; topical mydriatics were permitted. Group B also received antituberculous treatment for one year	No placebo, untreated or corticosteroid control group. Outcomes were compared before and after treatment; both study groups received curcumin	Clinical signs and symptoms at 12 weeks; recurrence and visual loss during three-year follow-up	All 18 patients receiving curcumin without antituberculous therapy were reported to improve; 12/14 (86%) receiving curcumin plus antituberculous therapy achieved remission. Recurrence occurred in 10/18 (55%) and 5/14 (36%), respectively. No comparative estimate, CI or formal statistical test was reported	Uncontrolled and unmasked; 21/53 participants (40%) did not complete treatment; small sample; poorly characterised and potentially mixed infectious and non-infectious etiologies; concomitant antituberculous treatment in one group; subjective and non-standardised response assessment; no adjustment for confounding; comparison with corticosteroid efficacy was indirect rather than contemporaneous
	Miserocchi et al. 2020 [79]; retrospective	27 patients aged 3-31 years with JIA-associated chronic	One Norflo tablet daily for 12 months, containing highly bioavailable	Baseline status and preceding recurrence	Anterior chamber flare measured clinically and by	Inactivity was achieved in 22/27 patients (81%).	Retrospective, small, uncontrolled and unmasked study;

	longitudinal before-after study	anterior uveitis and residual low-grade flare despite stable systemic immunosuppression; one eye per patient analysed	curcuminoids complexed as iPhytoone with ≥2% phosphatidylserine; the curcumin quantity per tablet was not reported. Existing systemic immunosuppression was continued	history; no concurrent placebo or untreated group	laser flare photometry; inactivity and recurrence over 12 months	Mean laser flare decreased from 34.3±8.8 to 11.8±13.1 photons/ms at 12 months; mean reduction 22.52 photos/ms (95% CI 14.63-30.40; $p<0.0001$). Three patients experienced flare-ups	selected patients had mild residual flare without anterior chamber cells; concomitant immunosuppression – including methotrexate and biologic therapy – was continued; natural disease fluctuation and regression to the mean cannot be excluded; flare may partly represent permanent blood-aqueous barrier damage; formulation dose was incompletely reported
	Dawczynski et al.2006 [81]; cross-sectional comparative study	24 patients with mixed anatomical uveitis: acute/first-episode uveitis (n=13: 6 anterior, 5 intermediate, 2 panuveitis) and chronic recurrent uveitis (n=11: 1 anterior, 4 intermediate, 4 posterior, 2 panuveitis)	Selenium and zinc concentrations measured in EDTA blood using atomic absorption spectroscopy; no participant was taking selenium or zinc supplements	Acute/first-episode uveitis versus chronic recurrent uveitis; no healthy control group	Blood selenium and zinc concentrations at a single assessment	Selenium was lower in chronic recurrent than acute uveitis (0.95±0.19 vs 1.34±0.13 µmol/L; $p<0.005$). Zinc was also lower, but the difference was not statistically significant (80.4±13.9 vs 90.1±6.8 µmol/L; $p=0.072$). Cis and adjusted estimates were not reported	Very small, cross-sectional study; no healthy comparator; chronic group was substantially older than the acute group (53.2 vs 30.0 years); heterogeneous and incompletely characterised uveitis; dietary intake was not assessed; several chronic patients received corticosteroids or ciclosporin; no adjustment for age, treatment,

							inflammatory burden or other confounders; cannot determine whether reduced selenium was a cause or consequence of chronic inflammation
**Sunlight & Vitamin D**	Rojas-Carabali et al.2024 [100]; systematic reviewer and meta-analysis of observational studies	11 studies of NIU; five included in the principal meta-analysis (354 cases and 5,728 controls); heterogeneous adult and pediatric NIU phenotypes	Circulating vitamin D concentration or vitamin D deficiency; definitions and timing of measurement varied	Participants without NIU or those with higher/sufficient vitamin D levels	NIU occurrence; secondary analyses considered inflammatory activity and related clinical variables	Vitamin D deficiency was associated with NIU: OR 2.04 (95% CI 1.55-2.68, $p<0.00001$). Serum vitamin D was lower in NIU cases: SMD -0.39 (95% CI -0.71 to -0.08; $p=0.0007$, $I^2=79\%$)	Based on observational studies; substantial heterogeneity in NIU phenotype, vitamin D thresholds, sampling timing and confounder adjustment; possible reverse causation and residual confounding; does not establish causality or supplementation efficacy
	Chiu et al.2020 [109]; prospective case-control study	151 adults with NIU recruited from three Australian centres: 74 with active and 77 with inactive anterior, intermediate, posterior or panuveitis; population reference data, n=594	Single serum 25-hydroxyvitamin D measurements; deficiency defined as <50nmol/L; questionnaire-reported vitamin D supplementation and sunlight exposure	Active vs inactive NIU; serum levels also compared with season- and location-stratified population estimates	Uveitis activity at assessment; no longitudinal follow-up	Mean 25(OH)D: 46 nmol/L in active vs 64 nmol/L in inactive NIU ($p<0.001$); OR for active uveitis per 1-nmol/L increase, 0.98 (95% CI 0.96-0.99). Supplement use was associated with inactivity ($p=0.026$); among vitamin D-deficient participants, greater weekday and weekend sunlight	Observational, essentially cross-sectional comparison; active and inactive groups were not matched; supplementation and sunlight were self-reported; possible reverse causation, including reduced sunlight exposure of active disease; heterogeneous NIU phenotypes and laboratory assays;

						exposure was associated with inactivity (both p=0.014)	residual confounding; does not establish supplementation efficiency
	Dadadci et al.2017 [110]; prospective observational case-control study	20 adults with active idiopathic or presumed HLA-B27-associated nongranulomatous AAU and 20 age- and sex-matched healthy controls in Turkey	Single serum 25-hydroxyvitamin D measurement using electrochemiluminescence	Healthy participants without ocular or systemic disease	Presence of active AAU; no prespecified longitudinal outcome	Mean 25(OH)D was 5.75±4.50 ng/mL in AAU vs 12.96±5.89 ng/mL in control (p<0.001); no adjusted estimate or CI reported	Very small, single-region study; cross-sectional measurement cannot establish temporality or causality; no multivariable adjustment; HLA-B27 was not tested; potential unmeasured differences in sunlight, diet and comorbidity; selected sample excluded smokers, obesity and recent supplement or alcohol use; assay may underestimate 25(OH)D
	Grotting et al.2017 [111].; retrospective case-control study	100 adults with idiopathic NIU anterior uveitis and 100 ophthalmic controls without ocular inflammation	Recorded serum total 25-hydroxyvitamin D; hypovitaminosis defined as below the reporting laboratory's reference range; timing examined relative to uveitis onset	Patients without uveitis who had recorded vitamin D levels	Presence of NIU anterior uveitis; subanalysis used measurements obtained before onset and without year before onset	Hypovitaminosis D: adjusted OR 2.96 (95% CI 1.60-5.50, p=0.001); when measured before onset, OR 3.25 (95% CI 1.52-6.95, p=0.002), and within one year before onset, OR 3.91 (1.52-10.10). Per 1-ng/mL increase in 25(OH)D: adjusted	Retrospective selection of patients with clinically obtained vitamin D measurements; relatively small temporal subgroups; possible selection and ascertainment bias; adjustment limited principally to age, sex and race/ethnicity; residual confounding

						OR 0.96 (95% CI 0.93-0.99, p=0.01)	by sunlight, diet, supplementation and health-related behavior; observational association does not establish causality or supplementation efficacy
	Sobrin et al.2018 [112]; retrospective matched case-control study using a US health-insurance claims database	558 adults with incident NIU and 2,790 controls matched 1:5 by age, sex, race/ethnicity and index date; anterior, intermediate, posterior and panuveitis codes included	Serum 25-hydroxyvitamin D measured within one year before NIU diagnosed; adequate ≥20 ng/mL vs low <20 ng/mL	Matched patients with a normal eye examination and no uveitis	Incident NIU identified by an initial and confirmatory diagnosis within 120 days	Adequate vs low vitamin D: adjusted OR 0.79 (95% CI 0.62-0.99, p=0.04); adjusted for geographic region and month of blood draw. Black subgroup: OR 0.49 (95% CI 0.30-0.80, p=0.004); white: OR 0.87 (95% CI 0.67-1.21, p=0.40); Hispanic: OR 0.60 (95% CI 0.33-1.10, p=0.10)	Claims-based diagnosis without medical-record confirmation or detailed NIU phenotype; vitamin D was measured in only 3%-5% of database members, creating potential selection bias; supplementation and smoking data unavailable; possible exposure misclassification and residual confounding; race-stratified findings may reflect limited subgroup power; observational design cannot establish causality or supplementation efficacy
	Susarla et al 2022. [113]; two sample Mendelian randomisatio	375 patients with combined NIU and/or noninfectious scleritis and 4,167 biobank controls	Genetic predisposition to lower lifelong 25-hydroxyvitamin D, using 75 genome-wide significant variants; analyses adjusted	Participants without recorded uveitis or scleritis	Susceptibility to combined NIU/noninfectious scleritis	Per SD decrease in log-transformed genetically predicted 25(OH)D: OR 2.16 (95% CI	Combined heterogeneous uveitis and scleritis outcome prevents NIU-specific

	n case-control study	without recorded uveitis or scleritis	for age, sex, ancestry principal components and multiple sclerosis			1.01-4.64, p=0.049). Excluding GC variants: OR 2.38 (95% CI 1.06-5.36, p=0.035). CYP2R1-restricted analysis: OR 6.42 (95% CI 3.19-12.89, $p=1.7x10^{-7}$)	interpretation; modest case number and borderline primary result; predominantly European-ancestry data; no independent replication; possible control misclassification; estimates depend on Mendelian-randomisation assumptions, including instrument validity and absence of pleiotropy; reflects lifelong genetic propensity and does not establish that supplementation prevents or treats NIU
	Shih et al.2025 [114]; retrospective analysis of the multicentre SITE cohort	Patients with chronic NIU anterior uveitis: 75 vitamin D users and 2,688 never-users; included idiopathic, spondyloarthropathy-associated and JIA-associated disease	Recorded use of any vitamin D supplement for ≤1 year; reported doses 400-5,000 IU/day; patients taking combined calcium and vitamin D were excluded	Patients who never used vitamin D	Remission, defined as inactive disease for ≥90 days without any ocular anti-inflammatory treatment; outcomes reported through 16 months	Adjusted remission incidence at 16 months: 28.0% with supplementation vs 13.5% without supplementation; adjusted HR 2.43 (95% CI 1.36-4.33, p=0.0027)	Nonrandomised retrospective study with a markedly imbalanced group size; baseline vitamin D status, indication for supplementation and adherence were unavailable; wide dose range and limited dose information; possible confounding by indication and health behavior; irregular follow-up and modified remission

							ascertainment; tertiary-centre cohort may represent more severe disease; residual confounding despite multivariable adjustment
**Obesity & body weight**	Ansari et al. 2018 [142]; retrospective population-based cohort study with a nested analysis among patients with diabetes	938,440 UK primary-care patients, including 48.584 with diabetes (3,273 type 1: 45,311 type 2); 253 acute uveitis episodes occurred among patients with diabetes	Glycemic control based on time-weighted HbA1c over the six-year study period: <7%, 7.0%-8.4%, 8.5%-11.3% or >11.3%	Patients with diabetes and HbA1c <7%; diabetes-vs-no diabetes analysis were also conducted	Recorded acute uveitis episodes from 2010-2015	Versus HbA1c <7%: OR 1.20 (95% CI 0.86-1.68, p=0.29) for 7.0%-8.4%; 1.57 (95% CI 1.05-2.33, p=0.03) for 8.5%-11.3%; and OR 4.72 (95% CI 2.58-8.65, p<0.001) for >11.3%. Diabetes vs no diabetes: type 1 OR 2.01 (95% CI 1.18-3.41, p-0.009); type 2 OR 1.23 (95% CI 1.05-1.44, p=0.01)	Retrospective primary-care database study; coding could not distinguish infectious from noninfectious uveitis or anatomical phenotypes; possible missed or miscoded secondary-care diagnosis and ascertainment bias; residual confounding despite adjustment; temporality between glycemic exposure and individual episodes Is imperfect; association does not demonstrate that improving glycemic control reduces uveitis risk or recurrence
	Pepin et al.2024 [143]; retrospective uncontrolled case series	89 patients with poorly controlled diabetes and otherwise idiopathic acute anterior uveitis (121 eyes); HbA1c ≥60 mmol/mol and negative HLAB-B27,	HbA1c recorded one year before, at presentation and one year after initial episode; HbA1c within ±3 months of recurrence where available	No external or well-controlled diabetic comparator; within-cohort comparison of recurrence	Clinical phenotype, resolution, recurrence and visual outcome; visual acuity reported to 12 months	Recurrence occurred in 22/121 eyes (18.2%). HbA1c one year after presentation was associated with recurrence (p=0.001); HbA1c	Retrospective, selected uncontrolled series; all participants already had both poor glycemic control and anterior uveitis, so incidence or

		syphilis serology, ACE and chest radiograph		according to HbA1c		before or at presentation was not associated (p=0.281 and p=0.493). No HR, CI or adjusted estimate reported	relative risk could not be assessed; no adjustment for confounding; possible selection and follow-up bias; recurrent eyes clustered within patients; predominantly Pacific population may limit generalizability; association does not demonstrate that improving HbA1c prevents recurrence
	Madhavan et al.2025 [136]; cross-sectional study	81 consecutive adults receiving adalimumab for NIU; anterior, intermediate, posterior and panuveitis; 90% bilateral; heterogeneous etiologies	BMI measured clinically; obesity defined as BMI ≥30 kg/m^2. Trough serum adalimumab and anti-adalimumab antibodies were measured using a chemiluminescent assay; antibody concentration > 10ng/mL was positive	Patients with vs without anti-adalimumab antibodies	Prevalence of anti-adalimumab antibodies and associations with BMI, trough drug concentration and clinical characteristics at a single assessment	Antibodies were detected in 5/81 patients (6.2%; 95% CI 2.7-13.6). Median BMI was 34.9 vs 28.4 kg/m^2 in patients with vs without antibodies (p=0.010); all five antibody-positive patients were obese. Median trough adalimumab concentration was 0.0 vs 5.0μg/mL (p=0.002)	Cross-sectional design cannot establish temporality or causality; only five patients had antibodies, preventing multivariable adjustment; BMI was available for only 48/81 patients, creating possible selection bias; heterogeneous NIU phenotypes and treatment regimens; concomitant immunomodulation, dosing interval and missed doses may affect immunogenicity and drug concentrations;

							patients who had previously stopped adalimumab may have been misses; assay differences limit comparison across studies
	Pichi et al.2025 [137]; single-centre retrospective cohort	40 children with JIA-associated chronic anterior uveitis treated with adalimumab; mean age 11.85 years; 65% female	BMI calculated using age- and sex-specific CDC growth charts. Adalimumab was administered every two weeks using weight-banded doing: 20mg if <30 kg and 40mg if ≥30 kg. Trough concentrations and anti-adalimumab antibodies were measured at 6 months	BMI 5th-85th percentile vs >85th percentile; responders vs nonresponders at 6 months	Clinical response and adalimumab trough concentration at 6 months; nonresponders were escalated to weekly dosing and reassessed at 12 months	Higher BMI correlated with poorer response (r=-0.634; p<0.001), lower trough concentration (r=-0.427, p=0.005) and higher antibody levels r=0.388, p=0.012). Response was 89.7% vs 25%, and mean through concentration was 5.7 vs 2.0 µg/mL, in the 5th -85th vs >85th percentile groups. All initial nonresponders achieved disease control after non-randomised escalation to weekly dosing	Small, single-centre retrospective study; no independent validation or multivariable adjustment reported; BMI, age, growth, body composition and weight-banded dosing are interrelated; possible effects of adherence, concomitant methotrexate, inflammatory burden and doing adequacy; weekly escalation was confined to initial nonresponders and was not randomised; does not demonstrate that weight reduction improved response
	Pichi et al. 2025 [138]; single-centre cross-sectional observational study	80 patients receiving adalimumab for NIU: anterior 51 (63.7%), intermediate 6 (7.5%), posterior 3 (3.7%) and panuveitis 20 (25.0%); 25 had isolated ocular	BMI at adalimumab initiation, analysed continuously and as normal weight, overweight, obese or morbidly obese. Adalimumab trough concentrations and anti-adalimumab antibodies were measured after a	Comparisons across BMI categories and between clinical responders and nonresponders	Adalimumab through concentration, anti-adalimumab antibodies and clinical response	BMI correlated inversely with adalimumab concentration (r=-0.408; p=0.007) and response (r=-0.40, p=0.009). In logistic regression using BMI as the sole	Small, single-cnetre, cross-sectional study with heterogeneous NIU phenotypes; timing of measurement varied substantially; analyses were not adjusted for age, sex,

		disease and 55 had an associated systemic inflammatory disease	mean of 17.9 months of treatment			predictor, BMI predicted response (p=0.017). Responders had higher mean through concentrations than nonresponders (11.9 vs 3.01 μg/mL, p=0.0001). BMI was not meaningfully associated with anti-adalimumab antibody concentration (r=0.027)	disease severity, comorbidities, adherence, dosing interval, inflammatory burden or concomitant immunomodulation; BMI is an imperfect measure of adiposity; reported BMI-category counts and proposed BMI thresholds were internally inconsistent; cannot establish causality or show that weight reduction restores response
	Mohan et al. 2025 [147]; multicentre retrospective propensity-score-matched HER cohort (TriNetX, 2006-2025)	Primary analysis: 258,026 patients prescribed a GLP-1RA and 258,026 matched controls; additional cohorts included patients with type 2 diabetes, without diabetes, and uses of other antihyperglycemic medications. Patients with previous uveitis were excluded	GLP-1RA exposure identified from prescription records; actual dispensing, adherence, duration and weight loss were not established	Matched patients without a GLP-1RA prescription; active comparators receiving metformin, insulin or an SGLT2 inhibitor	New ICD-coded noninfectious uveitis, including anterior uveitis, chorioretinal inflammation, panuveitis and retinal vasculitis; assessed at any time after prescription, with 1-, 3-, and 5-year sensitivity analyses	GLP-1RA vs matched non-use: RR 0.48 (0.46-0.51); with type 2 diabetes: RR 0.54 (0.51-0.58); without diabetes: RR 0.52 (0.46-0.59). GLP-1RA vs metformin: RR 0.58 (0.54-0.62); vs insulin: RR 0.57 (0.54-0.61); vs SGLT2 inhibitor: RR 1.17 (1.04-1.32). In a sensitivity analysis restricted to monotherapy, GLP-1RA use remained associated with lower incidence than metformin or	Retrospective prescription- and diagnosis-code-based study; prescriptions do not confirm medication use; residual imbalance in BMI and HbA1c remained after matching; possible residual confounding from metabolic disease severity, prescribing patterns, socioeconomic factors, treatment duration, adherence and concurrent immunomodulation; no confirmation

						insulin, but did not differ from SGLT2-inhibitor use	through clinical examination or imaging; no assessment of weight change, recurrence, inflammatory activity or visual outcomes; comparator-dependent findings; associations in patients with and without diabetes do not establish independence from metabolic effects or causality; no evidence for treating established NIU
**Sleep, psychological stress, exercise and alcohol**	Neti et al. 2021 [157]; single-centre 1:1 matched case-control study	78 adults with recurrent acute anterior uveitis: 39 with a recent attack and 39 with quiescent disease, matched by age, sex and HLA-B27 status	Self-reported exposures during the preceding month: sleep duration, weekly exercise duration, alcohol consumption and psychological stress measured using the five-item Srithanya Stress Test (ST-5)	Recent recurrent attack vs quiescent RAAU for at least six months without treatment	Association between preceding-month exposures and a recent RAAU attack	Adjusted associations: ST-5 score ≥3, OR 9.07 (95% CI 1.14-72.16, p=0.037); sleep <7 hours/day, OR 12.12 (95% CI 1.37-107.17, p=0.025). Unadjusted analyses found no statistically significant association for exercise duration, OR 0.86 per hour/week (95% CI 0.65-1.13, p=0.29), or alcohol consumption, OR 2.50 (95% CI 0.49-12.89, p=0.273)	Small single-centre Thai cohort; retrospective self-reported exposures susceptible to recall and reverse-causation bias; cases and controls were interviewed at different clinical stages; stress instrument may have limited international generalizability; alcohol was recorded without quantity or drinking pattern; sparse exposure counts and very wide confidence intervals; exercise and alcohol estimates were

							unadjusted; residual confounding and multiple comparisons; no intervention or prospective measurement
**Sleep**	Ezzahari et al.2013.[188]; cross-sectional study	30 Moroccan children with JIA aged 8-18 years; four had JIA-associated uveitis	Number of nocturnal awakenings recorded as part of JIA disease-activity assessment; no validated sleep-quality instrument was used	Children with JIA-associated uveitis (n=4) vs JIA without uveitis (n=26)	Presence of uveitis and its relationship with JIA activity, visual function and quality of life; single cross-sectional assessment	More awakenings reported in the uveitis group, but the association was not statistically significant (reported r=0.71; p=0.69); no CI or adjusted estimate reported	Only four participants had uveitis; severely underpowered; cross-sectional design; sleep was not the primary exposure and was not comprehensively or objectively assessed; no adjustment for pain, arthritis activity, medications or other confounders; cannot establish whether sleep disturbance influences uveitis
**Psychological Stress**	Berlinberg et al.2019 [207]; single-centre cross-sectional case-control study	80 adults with NIU of mixed anatomical phenotypes: 40 with inflammation active within the preceding 90 days and 40 with controlled disease; 40 age-matched controls without ocular disease	Perceived stress measured using the validated 10-item Perceived Stress Scale (PSS-10); diurnal salivary cortisol was also assessed in a subset	NIU vs nonuveitic controls; recently active vs controlled NIU	Perceived stress and salivary cortisol; single cross-sectional assessment	NIU was associated with a 4.3-point higher adjusted PSS-10 score than controls (95% CI 1.8-6.9, p=0.002), adjusted for age, sex, employment, education and income. Additional adjustment for systemic autoimmune disease: 3.7 points (0.9-6.4, p=0.01). Recently active vs controlled NIU: 1.0	Cross-sectional design prevents determining whether stress preceded or resulted from uveitis; self-reported stress susceptible to recall and reporting bias; single tertiary centre; controls did not have another chronic eye disease; possible residual confounding by pain, visual impairment, depression, treatment and

						point (-2.0 to 3.9, p=0.52). Salivary cortisol measures did not differ significantly	systemic disease; only 58.3% provided analysable cortisol samples; no evidence that stress reduction alters inflammation or recurrence
	Grajewski et al.2020 [208]; single-centre cross-sectional postal questionnaire study with retrospective clinical-record review	173 adults with anterior (n=85), intermediate (n=39), posterior (n=40) or panuveitis (n=9); 23 (13%) had infectious uveitis and 150 had noninfectious diagnosis. Two-year activity status was available for 125 patients	Perceived stress during the preceding four weeks and two years, assessed using the validated German PSQ-20	Clinically active vs inactive uveitis during the preceding two years; no separate healthy control group	Perceived stress according to uveitis activity; activity defined by anterior chamber or vitreous cell, or retinal/choroidal inflammation documented during the preceding two years	Mean two-year PSQ-20 score was higher with active than inactive disease: 0.48±0.22 vs 0.36±0.20 (p=0.005). In mixed regression, active disease was associated with a 4.45-point higher PSQ-20 score (95% CI 0.441-8.454, p=0.031). The association between activity and categorically increased stress was not statistically significant: OR 1.932 (0.888-4.2004, p=0.097)	Cross-sectional survey with retrospective two-year exposure and activity assessment; only 32.8% responded, creating substantial selection bias; included infectious and noninfectious uveitis without an NIU-specific activity analysis; temporal direction cannot be established; recall bias; heterogeneous phenotypes, treatments and activity severity; possible confounding by age, treatment, symptoms and visual impairment; no evidence that stress reduction changes ocular outcomes
	Maca et al.2011 [209]; single-centre cross-	171 adults with recurrent HLA-B27-associated acute anterior uveitis; 13 (7.6%) reported an	Depression and coping assessed using the Beck Depression Inventory, Freiburg Questionnaire on Coping with Illness and	Patients vs healthy controls for depression and coping; patients	Depressive symptoms, coping patterns and patient-perceived triggers of previous	Mean depression score was higher in patients that controls: 8.7±7.0 vs 4.4±4.3 (p<0.001);	Cross-sectional design; distress preceding attacks was based entirely on retrospective patient

	sectional postal survey	active recurrence when surveyed; 55 age- and ethnicity-matched healthy controls provided normative psychological data	Stress Coping Inventory. Patients retrospectively reported whether psychological distress or specific life events had preceded previous attacks	reporting vs not reporting distress before previous recurrences	recurrences; single survey with retrospective reporting over a mean disease duration of 14.3 years	31.6% had depressive scores above normal range. Overall, 57.9% reported distress before previous attacks and 34.5% identified specific life events. No adjusted prospective estimate of recurrence risk was reported	attribution; substantial recall and attribution bias; recurrence history was self-reported; 45% survey response with possible selection bias; no prospective exposure measurement or objective comparison of recurrence rates; no multivariable adjustment for disease burden, pain, systemic spondyloarthritis, treatment or socioeconomic factors; cannot establish that distress triggers recurrence
	Shantha et al. 2026 [215]; single-centre, single-masked, waitlist-controlled RCT	100 adults with active or inactive NIU and at least mild anxiety or depression; intervention n=49, control n=51; mixed anatomical subtypes	Calm Health digital mindfulness program, recommended ≥10 min/day for 8 weeks	Waitlist control receiving no new mindfulness intervention during the study	Anxiety (GAD-7; primary), depression (PHD-9), perceived stress (PSS-10), and vision-related quality of life (NEI VFQ-25) at 8 weeks	Baseline-adjusted between-group differences: GAD-7 score -1.70(-3.17 to -0.23, p=0.02); PHQ-9 score -1.90 (-3.04 to -0.76, p=0.001); PSS-10 score -3.69 (-6.00 to -1.37, p=0.002); NEI VFQ-25 score 1.98 (-0.90 to 4.86, p=0.18)	Short follow-up; 30% did not complete the primary assessment; waitlist rather than active control; participants were unmasked and outcomes were self-reported; single tertiary centre with English speaking smartphone users; insufficient power for uveitis-subtype analyses. Ocular inflammatory activity, recurrence and

							corticosteroid exposure were not evaluated as outcomes

† Principal human studies informing the narrative synthesis; formal risk-of-bias grading was not performed. The most fully adjusted estimate is reported where available; unadjusted estimates are labelled. Separate rows indicate distinct exposure-comparator or outcome analyses.

‡ Abbreviations: BMI, body mass index; CI, confidence interval; CME, cystoid macular edema; ES, effect size; HR, hazard ratio; NIU, non-infectious uveitis; NR, not reported; OR, odds ratio; POHS, presumed ocular histoplasmosis syndrome; RAAU, recurrent acute anterior uveitis; RR, relative risk; 25(OH)D, 25-hydroxyvitamin D.
